# Demography and dynamics of giant kelp cohorts across four decades: Lessons for conservation and resilience planning

**DOI:** 10.1002/eap.70181

**Published:** 2026-01-28

**Authors:** P. Edward Parnell, Cleridy E. Lennert‐Cody, Lydia B. Ladah, Kristin L. Riser, Brenna Bulach, James J. Leichter, Ami K. Latker, Stephen C. Schroeter, Paul K. Dayton

**Affiliations:** ^1^ Integrative Oceanography Division, Scripps Institution of Oceanography University of California, San Diego San Diego California USA; ^2^ Department Biological Oceanography CICESE Ensenada Baja California Mexico; ^3^ City of San Diego Public Utilities Department San Diego California USA; ^4^ University of California Santa Barbara, Marine Science Institute Santa Barbara California USA

**Keywords:** climate change, cohort, conservation, demography, kelp, long‐term ecological research, *Macrocystis pyrifera*, management, marine heat waves, monitoring, sea urchins, southern California, understory

## Abstract

Kelp forests throughout many temperate zones are in decline due to various human stressors, chiefly marine warming. Conservation measures including restoration are presently of great interest and focus on both historical and novel methodologies. Of paramount importance for these efforts is an understanding of the mechanics of kelp decline to identify the factors and triggers leading to stepwise declines and thus support the development and spatial prioritization of strategic intervention to facilitate resilience. Here, we utilized a unique dataset documenting the demographic dynamics of giant kelp, *Macrocystis pyrifera*, in response to multiple disturbances across >40 years off San Diego (California, USA). The recruitment and life history of >14,000 individuals were used to evaluate cohort structure, size, and longevity forced by algal community structure and disturbance. Cohort dynamics varied spatially by depth and study subregion, thus aiding identification of areas to prioritize intervention to foster resilience. Five algal assemblages were characterized providing context for cohort dynamics in response to physical disturbances and sea urchin grazing. A trend of decreasing cohort size and resilience was observed over time accentuated by the marine heat wave of 2014–2015 (MHW) after which competition with understory canopies increasingly interfered with giant kelp cohort development and plant size structure. Cohort recruitment ranged on a continuum from discrete (“pulsed”) to more gradual (“trickled”) episodes. Pulsed cohorts mainly produced single cohort‐dominated age stands punctuated by major disturbances. Pulsed events were more common than trickled recruitment, especially at deeper sites. Trickled cohorts resulted in relatively mixed age stands, especially when individual cohorts overlapped within sites. Trickled recruitment increased over time as understory dominance increased. Cohort longevity was highly variable among sites and among cohorts within a site, with high first‐year mortality mostly due to warming, waves, or their combination. Longevity was inversely related to temperature and sea urchin density, and was greatest at deeper sites, especially after the MHW. The downward trend of single cohort dominance and individual plant size over time and its step downward after the MHW suggest that deeper areas should be prioritized for restoration. Regardless, understory canopies will increasingly dominate Southern California with continued warming.

## INTRODUCTION

A central question in ecology addresses understanding the processes that determine the distribution and abundance of populations in both time and space (Krebs et al., 2024). Longer term demographic studies of populations can offer a robust understanding of the processes that shape overall fitness, as well as the general dynamics of the community under study (Koons et al., 2016; McDonald et al., 2017). The life cycles of any population evolve within the context of a complex web of ecosystem processes that define demographic patterns within the larger system, which can change dramatically at various time scales and are prime determinants of the processes influencing natural selection (Wilbur, 1980). Understanding the demographic properties of a population over various spatiotemporal scales is therefore critical to an integrative understanding of the ecology, evolution, and conservation of any species (Rees et al., 2001).

The giant kelp, *Macrocystis pyrifera*, is a conspicuous and charismatic foundation species in many north Pacific coastal habitats (Dayton et al., 1992) and is the most studied and best‐known kelp species in the world. It forms large coastal forests along the northeast Pacific Ocean from Baja California, Mexico to Alaska, USA, and most suitable habitats within the Southern Ocean (Wernberg et al., 2018). Demographic responses of giant kelp result from both biotic and abiotic ecological processes (Dayton et al., 1984; Layton et al., 2019), and different populations can function differently due to their inherent degree of adaptation that can affect their demography and population growth potential (Graham et al., 2007). Giant kelp lives in an environment subject to a wide range of physical disturbances across time and space which effectively negate its ability to achieve population stability and a stable age distribution (Dayton et al., 1999; Dayton & Tegner, 1984; Tegner et al., 1997), highlighting the importance of longer term demographic studies in understanding its population ecology.

Although it is well known that abiotic or density‐independent disturbances such as storm waves (Seymour et al., 1989) and marine warming profoundly affect giant kelp populations by causing mass mortality, the demographic response of recovering giant kelp populations is not well documented (though see Rosenthal et al., 1974) and varies in time and space. A host of factors contribute to such complexity, including variability in the amplitude of disturbance across environmental gradients, patch dynamics (Dayton et al., 1984, 1992), seascape (Parnell, 2015), competition for space with other species (Arkema et al., 2009), biophysical factors controlling recruitment and growth (Dayton et al., 1999), the local hysteresis of disturbance and recovery supporting algal composition prior to and after large disturbances (Carnell & Keough, 2020), and connectivity with other populations (Alberto et al., 2010).

Considering the various challenges of marine conservation and restoration, understanding where to focus effort is critical (Fraschetti et al., 2021). This novel long‐term cohort analysis of a declining foundational species across numerous sites and depths informs conservation and restoration practices by identifying areas with the highest potential for recovery and the strategy that would have the greatest chance of success across different disturbances. Given both the large spatial and temporal scale of this study, our data not only provide insights into the immediate effects of these stressors but also facilitate an assessment of recovery times over a range of spatial scales. This information is essential in evaluating whether adaptive management (i.e., remediation) is necessary and if so, the nature and likely success of such remedial measures. Here, we present demographic data characterizing *M. pyrifera* populations throughout San Diego County (southern California, USA). The detailed analysis of cohort survivorship over 20–40 years in many different microhabitats offers a fine‐scale understanding of the selective strengths of the various biotic and abiotic conditions, and how giant kelp demography has responded to these conditions over time. We present data on adult recruitment and mortality as well as the individual lifespans of plants from these southern California forests that are among the largest along the US west coast. These data are integrated with habitat characteristics including substratum composition and topography from depths ranging from 8 to 21 m, algal assemblage characterization, and changing oceanographic climate patterns over the 20–40‐year period, describing the recruitment and mortality of plants within specific cohorts subjected to various stressors including marine heat waves, storm wave events, overgrazing by sea urchins, and competition with understory canopies.

## METHODS

### Setting

**FIGURE 1:**
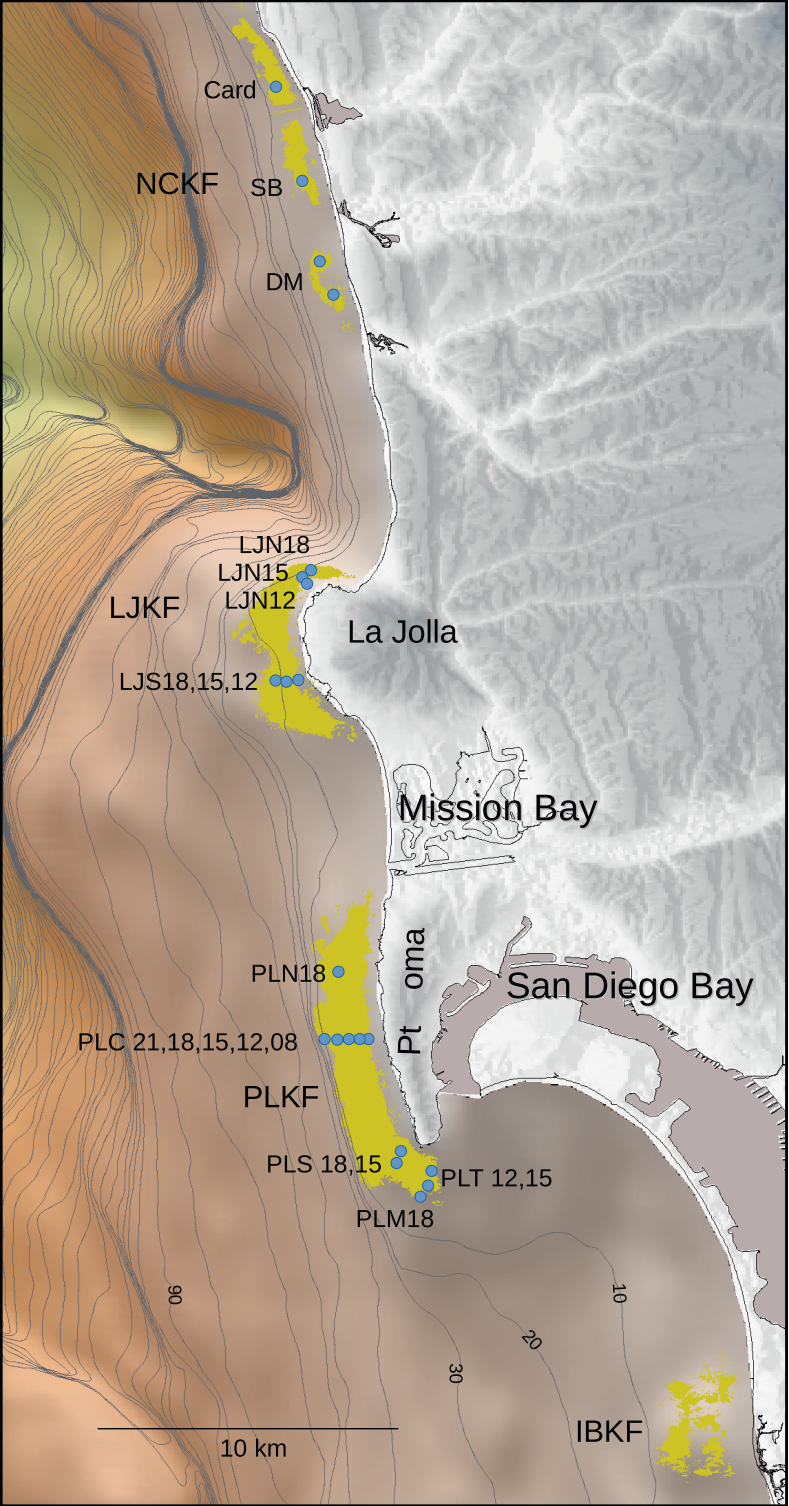
Map of kelp forest study sites off San Diego, CA. Gold shading represents areas where *Macrocystis pyrifera* canopy was observed from 1967 to 1999 in aerial photographs. Contours in meters. NCKF, LJKF, PLKF, and IBKF refer to the kelp forests off North County, La Jolla, Pt. Loma, and Imperial Beach, respectively.

The kelp forests off San Diego reflect unique hard bottom substrata (Emery, 1960) and include the two largest kelp forests off the US west coast and smaller fragmented beds on low relief bedrock (Figure 1). The Pt. Loma and La Jolla kelp forests are supported by two Cretaceous sandstone shelves shallow enough for kelp to grow contiguously up to 2 km offshore (Parnell, 2015). The smaller fragmented forests in northern San Diego County are shallower in extent and are surrounded by unconsolidated sediments.

The kelp program off San Diego began in the 1970s (Dayton et al., 1992) and continues at present. Five permanent study sites were established in 1983. Fifteen additional sites have been added since then spanning ~45 km of coastline (see Table 1 for site information and habitat descriptions).

**TABLE 1:** Study site information.

Site	Depth (m)	Year est.	Physiography
Card	17	2006	Low relief mudstone, small patch reef surrounded by sand
SB	16	2006	Low relief mudstone, small patch reef surrounded by sand
DM	16	2007	Low relief mudstone with sumps subject to sand inundation, small patch reef surrounded by sand
LJN12	12	2004	Mixed bedrock with few low relief reefs and sand channels
LJN15	15	2004	Mixed bedrock with small (<20 cm) ledges and sand channels
LJN18	18	2004	Mixed low relief bedrock flanked by large reef systems north and south within 15 m
LJS12	12	2004	Mixed sandstone bedrock with <35 cm of sharp vertical relief reefs and sand channels
LJS15	15	1992	Mixed low relief bedrock with small ledges (<20 cm) and sand channels
LJS18	18	2004	Low relief sandstone flanked by bedrock reefs and boulders on all sides
PLN18	18	1983	Low relief sandstone with small (<25 cm) ledges
PLC08	8	1997	Low relief sandstone with small (<25 cm) ledges
PLC12	12	1983	Low relief sandstone with widely dispersed small (<75 cm diameter) boulders
PLC15	15	1983	Low relief sandstone with small (<25 cm) ledges, large overhang, and boulder reef 15 m to west
PLC18	18	1983	Low relief sandstone with small bedrock reefs (<1 m) between transect lines; site is flanked by larger reef systems on all sides
PLC21	21	1995	Low relief sandstone on <1° slope dipping southwest
PLS15	15	1992	Low relief mudstone with small (<50 cm) reefs mostly between transect lines; no sand
PLS18	18	1983	Low relief mudstone heavily bio eroded by pholads, small (<30 cm) ledges and boulders scattered throughout mostly between lines, flanked by reef ridge system 20 m to north, adjacent to an urchin barren
PLT12	12	1997	Low relief mudstone with small (<25 cm) ledges, occasional coarse silt and very fine sand in sump channels; large ridge reefs flank site within 25 m
PLT15	15	1997	Low relief mudstone with coarse silt, few small ledges (<20 cm)
PLM18	18	1996	Low relief mudstone with coarse silt in small sumps, few small ledges and boulders (both <25 cm) flanked by ridge reefs within 20 m east and west

Four permanent leaded transect lines each 25 m in length and separated by 8–10 m are oriented perpendicular to shore at each site. Each transect is censused for algae (quarterly) within 2 m on each side. Transects are divided into 5‐m sections yielding ten 5 × 2 m quadrats which compose individual sampling units nested within transect line and study site encompassing 400 m^2^ of bottom per site. All algae in the order Laminariales are enumerated and the fractional cover of turf algae is visually estimated within each quadrat. Turf algal categories include articulated coralline, crustose coralline, and foliose Rhodophytes. Turf Phaeophytes include all Dictyotales pooled with the exception of *Dictyopteris undulata* and *Dictyota binghamiae*, which are estimated individually. The percent cover of *Cystoseira osmundacea* is also estimated within each quadrat. Life stages of *M. pyrifera* include juveniles (all stages <1 m off bottom and <2 stipes), pre‐adults (plants which have undergone primary dichotomous branching and are taller than 1 m), and adults (plants ≥4 stipes). Adults are mapped upon recruitment to adulthood, and their stipes are counted until they die.

### Bottom temperature

Bottom temperatures have been recorded at the central Pt. Loma 15‐m study site (PLC15) since 1983 at 3‐h intervals. Instrumentation has evolved over time beginning with Ryan Thermographs (1983–1990), Ryan RTM Tempmentor thermographs (1990–1999), and Onset Tidbits (1999–present). Data gaps were <1 month in duration and were linearly interpolated using R (R Core Team, 2021).

### Waves

A hindcast of wave height and period (*H*_*s*_ and *T*_*p*_) at the CDIP Torrey Pines Outer Buoy (Station #100) was used to model the effects of wave disturbance on algal stands over time. The hindcast (O'Reilly et al., 2016) results from a combination of the NOAA CFSR2 (3‐h timesteps) wind‐wave model hindcast (1979–present) and direct observations from buoy #100 (2001–present). CFSR2 hindcast data were decimated to weekly maximum *H*_*s*_ and coincident wave period (*T*) which were then converted to wave energy (Appendix S1: Equation S1). *H*_*s*_ observed at CDIP Station #100 characterizes the wave energy along the entire San Diego coastal shelf (O'Reilly, personal communication).

### Analyses

#### Algal assemblages

Algal transect data were clustered into assemblages using the “clara” (Kaufman & Rousseeuw, 2009) function (distance metric = Manhattan) in the “cluster” R library (Maechler, 2018). The resulting clusters represent the dominant algal states within each quadrat over time. All transect data from all sites were clustered utilizing quadrat data over the periods data were available for each site. Species data were first scaled then combined into categories of canopy guilds. *M. pyrifera* adults and juveniles were included as life stages in the cluster analysis. Juveniles included the sum of life stages prior to adulthood. Plants were considered adults after they reached their second primary dichotomy (≥4 stipes). Stipe counts were included as a separate algal category. Other categories in the cluster analysis included stipitate, laminate, brown turf, and articulated coralline algal guilds. *Desmarestia ligulata*, a low growing successional pioneer indicative of post disturbance conditions (Reed & Foster, 1984), was included as a separate category in the cluster analysis. Stipitates included *Pterygophora californica* and *Eisenia arborea*; laminates included *Laminaria farlowii* and *Agarum fimbriatum*; brown turfs included all Dictyotales and *C. osmundacea*; and articulated corallines included all low growing coralline turf species. *C. osmundacea* was included as a brown turf as it mainly exists as a low (<15 cm) growing turf‐like species in southern California extending further off the bottom only when reproductive in spring. The number of clusters utilized in subsequent analyses was chosen as a trade‐off between the gap statistical method of optimal cluster estimation (“fviz_nbclust” function in the R library “factoextra”; Kassambara, 2016) and expert knowledge of characteristic algal states. Algal states were then characterized as the fraction of quadrats in each cluster over time at each site.

#### Cohorts

Cohorts were first identified as periods of adult recruitment termed “cohort windows.” Cohorts were distinguished as pulses of adult recruitment typically lasting less than 18 months that were discrete from one another by at least one year. Parameters for each cohort were then characterized for further analysis to explore patterns and examine forcing. Parameters included the number of plants in each cohort, the number of juveniles within one year of cohort onset, mean lifespan, the number of adults observed only once (“singles”), Kaplan–Meier derived quantiles of survivorship (R library “survival”—Therneau, 2020), the number of *M. pyrifera* adults existing within one year prior to cohort onset (potential spore provisioning), understory and turf cover within one year of cohort onset, scaled algal guilds prior to and during cohort lifetime, and pooled understory algae (all algae except *M. pyrifera*) prior to and during cohort lifetime. Additional factors included depth, decade, and densities of the two species of sea urchins known to overgraze kelp forests off California (*Strongylocentrotus purpuratus* and *Mesocentrotus franciscanus*). Cohorts were classified as pulsed if at least 80% of plants recruited to adulthood within the first year since the appearance of the first plant in that cohort and whose adult recruitment was distinct from other cohorts by at least one year.

#### Cohort parameter modeling

Factor analysis was conducted to examine relationships among cohort parameters for inclusion in further analyses (R function “factanal,” rotation = “varimax,” scores = “regression”). The first two factors and their individual cohort parameter components were then used as dependent variables in generalized additive model (GAM) analyses. Random forest analysis (R library “randomForest”—Liaw & Wiener, 2002) was then used to identify cohort parameters that were potentially important as independent variables for GAM analyses. GAM models (R library “mgcv,” family = negative binomial, method = generalized cross validation, double penalty shrinkage) were run for the first two factors resulting from the factor analysis and the individual cohort parameters that most contributed to those factors. The two models that explained the most deviance for each axis from the factor analysis were chosen. Distributional assumptions were assessed and the appropriateness of basis function number for each GAM was checked using “gam.check” (Wood, 2017).

## RESULTS

### Physical forcing

**FIGURE 2:**
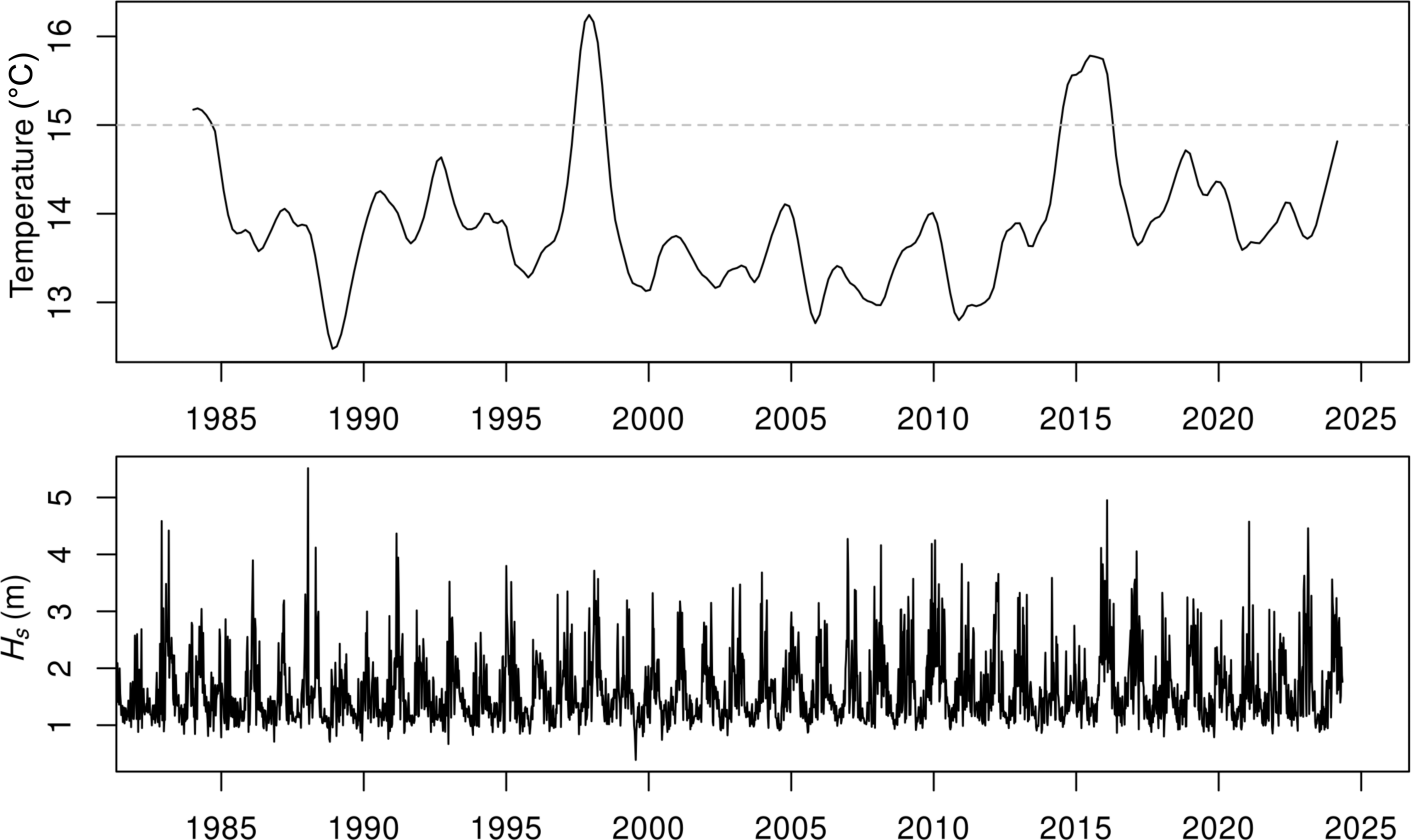
Top: Seasonally detrended bottom temperature at the PLC15 study site, hashed line indicates the temperature above which [NO_3_ + NO_2_] is considered limiting (Parnell et al., 2010). Bottom: Hindcasted significant wave height at CDIP station #100 (see text).

Several episodes of mortality due to physical disturbances were observed, including marine heat waves and damaging storm waves. El Niño events that caused extensive mortality of *M. pyrifera* occurred in 1983/1984, 1997/1998, and 2015/2016 (Figure 2). The 2015/2016 El Niño was preceded by a marine heat wave emanating from the NE Pacific originally termed the “Blob” (Bond et al., 2015). This marine heat wave merged with the strong El Niño of 2015/2016 and together decimated *M. pyrifera* off San Diego County and throughout northern California (Rogers‐Bennett & Catton, 2019). The most damaging wave events included the storms associated with the 1982/1983 El Niño, the 200‐year storm of 1988 (Seymour et al., 1989), the El Niño of 2016, and large storms of 1992, 2021, and 2023. The 1988 storm was immediately followed by cool La Niña conditions leading to rapid recovery of *M. pyrifera* (Dayton et al., 1999).

### Algal assemblages

**FIGURE 3:**
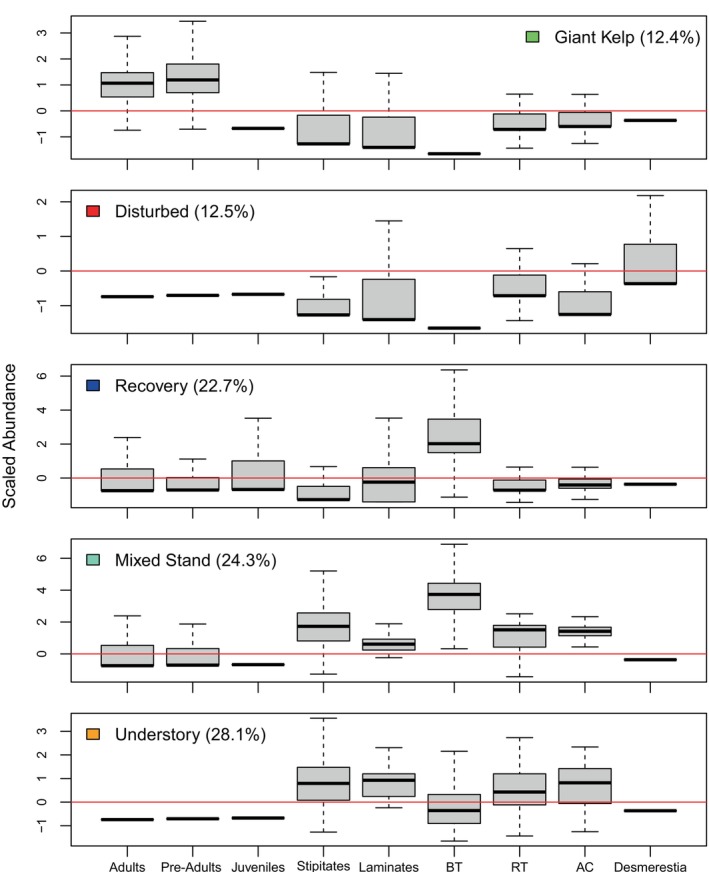
Boxplots of algal cluster composition. Adult, Stipe, and Juvenile categories indicate scaled densities of *Macrocystis pyrifera*. BT, RT, and AC represent brown turf, red turf, and articulated coralline algae. See text for species included as stipitates and laminates. Percentages of quadrats in each category are shown in parentheses. Boxes indicate the 25th and 75th percentiles, brackets indicate 5th and 95th percentiles, and dark lines indicate medians.

Algal communities exhibited five different assemblages (cf. states), represented by five clusters (Figure 3, Appendix S1: Figures S1–S5). The optimal number of clusters estimated using the gap method was four. However, based on extensive observation, four clusters were deemed inadequate from a natural history perspective since a cluster representing a mixed algal stand (balanced densities of canopy and understory species) was not included. Therefore, cluster analysis was repeated to include five clusters. Cluster 1 represents a well‐developed stand of *M. pyrifera* (hereafter “Kelp”). Cluster 2 indicates a disturbed state with sparse giant kelp coverage and moderate‐to‐high densities of the ephemeral early successional *D. ligulata* (“Disturbed”). Post‐disturbance recovery is represented by Cluster 3 (“Recovery”), which has the greatest densities of juvenile *M. pyrifera* and brown turf algae. Brown turf algae quickly responded to increased levels of benthic light that occurred after collapses of the *M. pyrifera* canopy. Cluster 4 indicates a mixed stand of *M. pyrifera* and understory species (“Mixed”). Cluster 5 represents a stand dominated by understory species (“Understory”).

**FIGURE 4:**
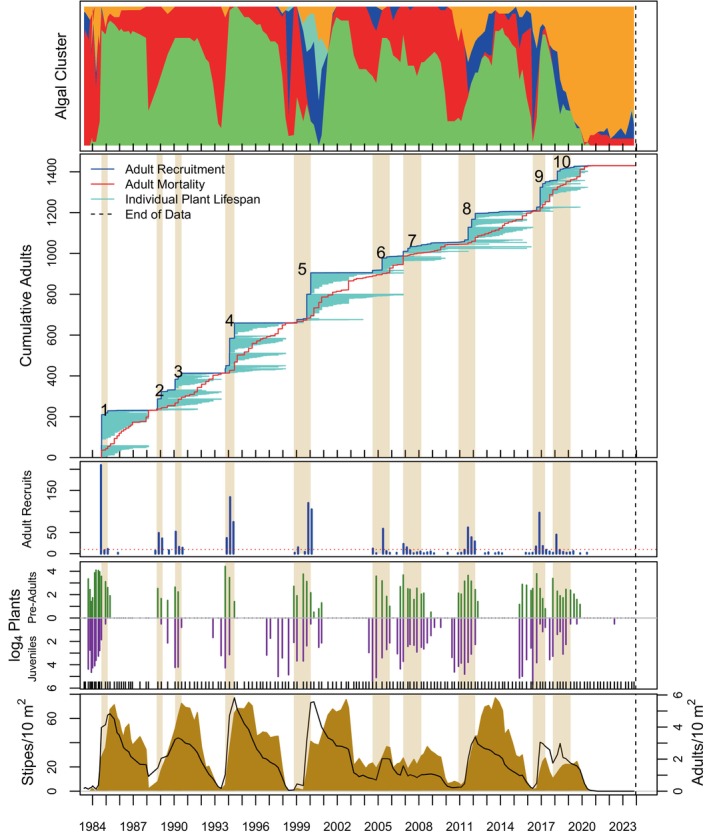
Timelines of algal cluster composition (top panel). Cumulative adult *Macrocystis pyrifera* recruitment (blue) and mortality (red) and individual plant lifespans (light blue lines) are shown in panel 2. Also shown are adult *M. pyrifera* recruits (panel 3), juvenile and pre‐adult life stages of *M. pyrifera* (log4 counts—panel 4), and stipe (black curves) and adult densities (gold areas) of *M. pyrifera* (panel 5) at PLN18. Gold bars running through plots 2–5 indicate cohort recruitment windows; numbers indicate cohort number.

**FIGURE 5:**
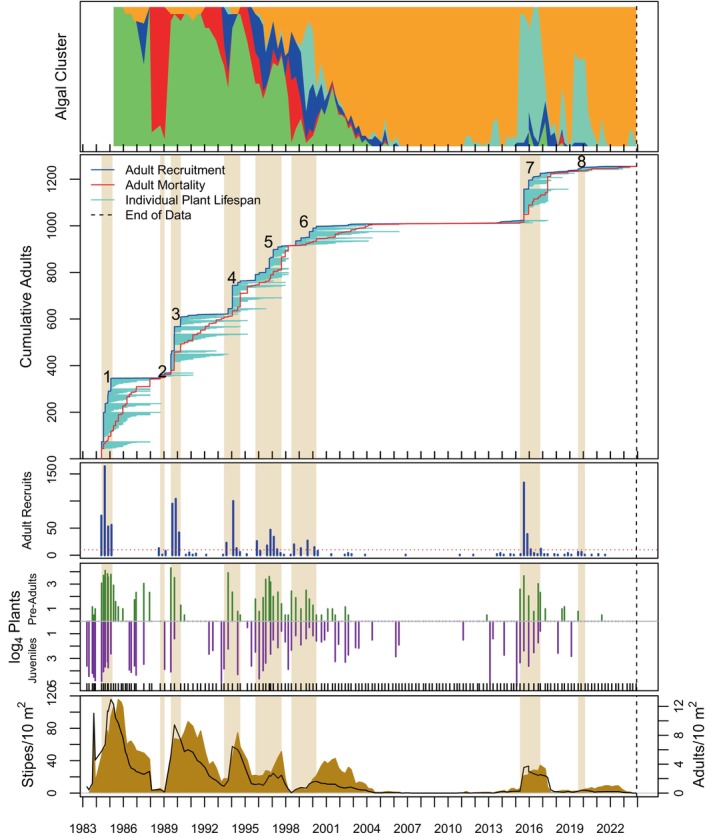
Same as Figure 4 but for the PLC12 study site.

**FIGURE 6:**
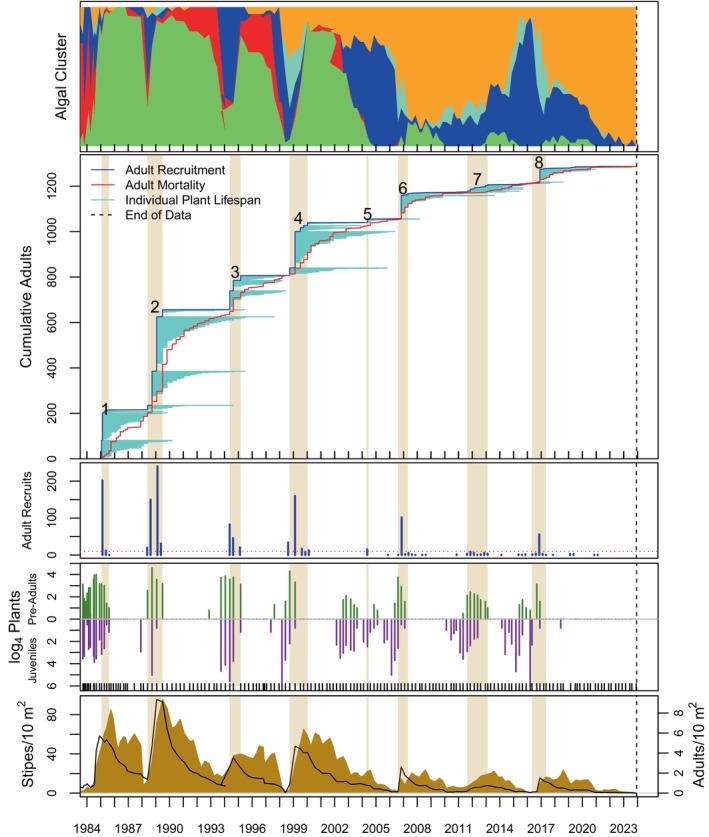
Same as Figure 4 but for the PLC15 study site.

**FIGURE 7:**
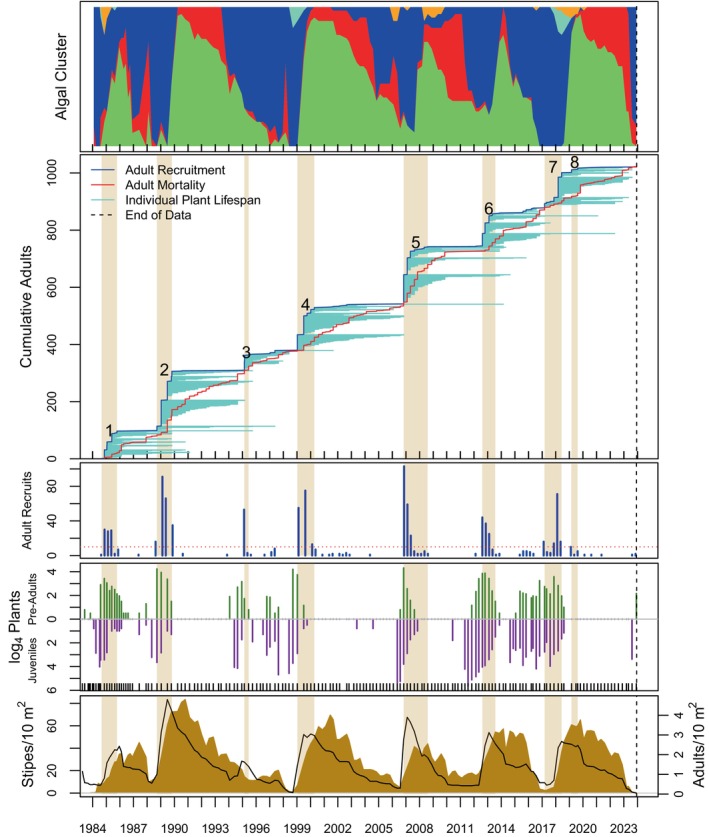
Same as Figure 4 but for the PLC18 study site.

**FIGURE 8:**
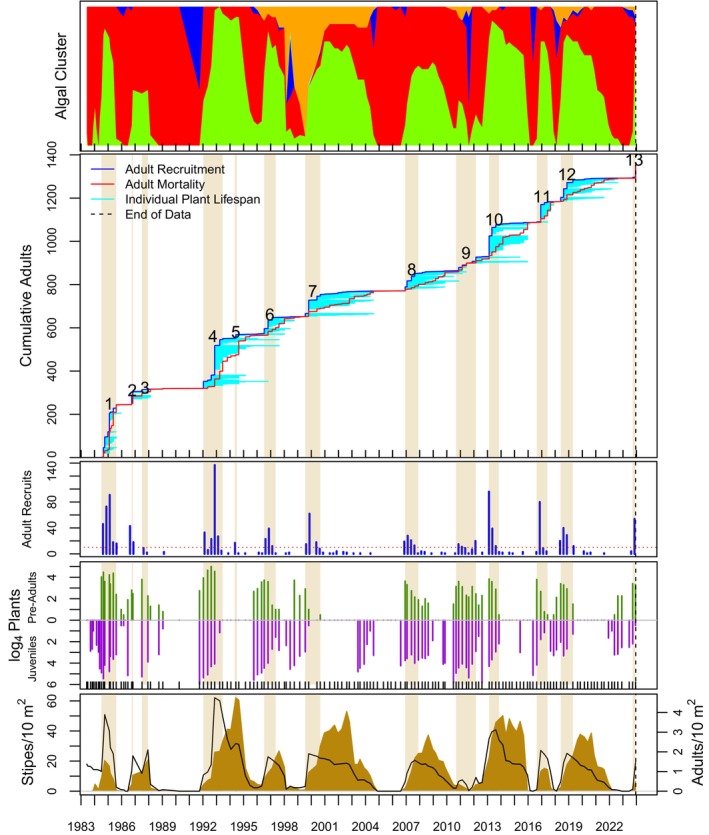
Same as Figure 4 but for the PLS18 study site.

Algal stand composition was dynamic over time and among sites (top panels of Figures 4, 5, 6, 7, 8, Appendix S1: Figures S6–S20). Three major patterns emerged from cluster analysis. First, *M. pyrifera* domination waxed and waned in discrete pulses mostly associated with major disturbances or cohort thinning. In this pattern, declines of *M. pyrifera* were followed by short periods (months or less) of a disturbed state immediately followed by the recovery state. This pattern is consistent with other studies (Dayton et al., 1999; Reed et al., 2011) in which major disturbances such as El Niño events or unusually strong wave events decimate *M. pyrifera* canopy leading to increased benthic light levels. This facilitates pulses of giant kelp recruitment, which manifest as discrete cohorts (Tegner et al., 1997). The magnitude of this response appears greater with increasing depth, which is evident by contrasting the 18‐ and 21‐m sites off central Pt. Loma with shallower sites.

The second pattern included phase shifts in algal state (heretofore characterized as Kelp, Mixed, Disturbed, Understory, Recovery) that persisted for years after physical disturbances including the 1997 El Niño event, the MHW of 2014–2015, the wave events of the early 2020s, and the 1988 storm wave event. These shifts in algal state based on cluster analysis are summarized in Table 2. Most sites that were dominated by the Kelp state at the beginning of their respective time series shifted to the Understory state by either the 2014–2016 MHW or the wave events and warming of the early 2020s. Presently, 12 sites are dominated by the Understory state (Card, LJN12, LJN15, LJS12, LJS15, PLN18, PLC12, PLC15, PLS15, PLT12, PLT15, and PLM18). Three sites are presently in the Disturbed state (DM, LJS18, and PLS18), and two appear to be in the Recovery state (LJS18 and PLC21). A few phase reversals occurred absent prior disturbance, thus indicating that phase reversals occur for reasons other than extreme physical disturbance. However, sites in the Kelp‐dominated state have decreased over the last decade with none at present.

**TABLE 2:** Summary of algal state shifts based on cluster analysis.

Algal cluster state switch	Sites
Kelp to understory	Card, LJS15, LJS18, PLN18, PLC12, PLS15, PLS18, PLT12, PLT15, PLM18
Understory to kelp	PLS15, PLS18
Kelp to disturbed	DM, PLS18
Understory to recovery	LJN18
Kelp to protracted recovery	SB, PLC21
Recovery to understory	PLC15, PLC21
Mixed to understory	LJN12, LJN15, LJN18, LJS12, LJS15, PLC08, PLC12
Understory to mixed	LJN15, LJS15, PLC12

The third general pattern was that sites within subregions of San Diego County exhibited similar algal assemblage states over time. The mixed algal assemblage was most common in the northern La Jolla kelp forest, while the southern Pt. Loma sites exhibited the most frequently disturbed assemblages. Presently, many sites have become dominated by understory, thus partially subsuming this subregional similarity pattern.

### Cohorts

Totally 14,166 adult *M. pyrifera* plants were followed from recruitment as adults (≥4 stipes) until their death at all sites over time. Of these, 13,186 plants were attributed to 149 adult cohorts (see Appendix S1: Table S1 for a list of cohort parameters). Ten cohorts were still alive as of this analysis, thus leaving 139 cohorts available for GAM analyses. Cohorts were evident as pulses of recruitment (see Figures 4, 5, 6, 7, 8 for long‐term sites, Appendix S1: Figures S6–S20 for the remaining sites).

**FIGURE 9:**
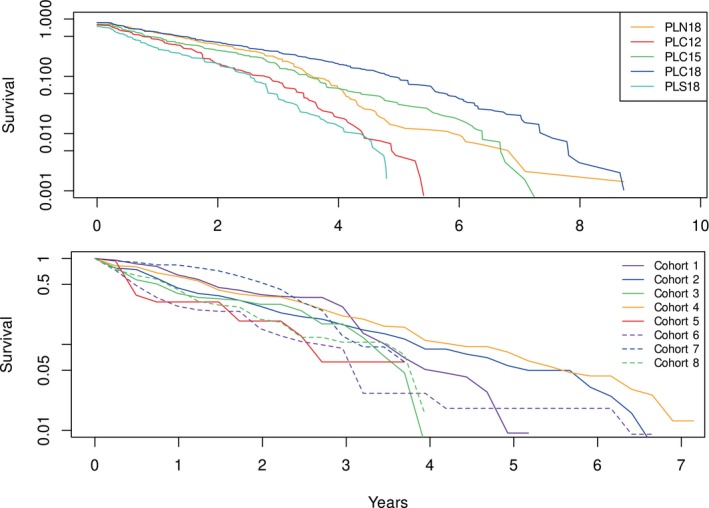
Survival curves of *Macrocystis pyrifera* at the long‐term sites (1984–2023). Survival curves for each study site represent survival of all cohorts pooled at each site (top). Survival curves for individual cohorts at the PLC15 study site (bottom).

Survival was highly variable among sites and cohorts within a site (Appendix S1: Table S1). The longest‐lived plant survived ~9.7 years (Cohort 6 at PLC18). For all plants pooled among all sites, survival of the 25th, 50th, 75th, and 95th percentiles were 0.21, 0.73, 1.74, and 3.9 years, respectively. A comparison of the survival of all plants pooled within each long‐term site (PLN18, PLC12, PLC15, PLC18, and PLS18—Kaplan–Meier analysis; Figure 9, top panel) indicates the deepest sites (PLC18 and PLN18) had the greatest survivorship except for PLS18, which has experienced episodes of mass mortality due to sea urchin overgrazing due to its proximity to a historically resilient sea urchin barren (Parnell, 2015). Survival among the central Pt. Loma sites exhibited a clear gradient of increasing survival with increasing depth. However, despite its greater depth, survival at the PLS18 site was less than that of the shallow 12‐m central Pt. Loma site.

Cohort survival within a site was highly variable (Figure 9; bottom panel). Sudden mortality for several cohorts was evident from the plots in Figures 4, 5, 6, 7, 8 and Appendix S1: S6–S20. Most sudden cohort mortality was due to extreme warming or storm wave events, although urchin grazing and amphipod infestation caused a few cohorts to decline precipitously. A lack of juvenile *M. pyrifera* recruitment within 3 months of the death of at least 95% of the plants in the preceding cohort was mostly observed where understory species increased to dominance or near dominance.

Giant kelp cohorts exhibited three general characteristics across time and space. First, some cohorts developed gradually (def., Trickled cohorts; Table 3), whereas others appeared abruptly as large pulses of adult recruitment (def., Pulsed cohorts). Pulsed cohorts typically appeared after major disturbances when algal stands, including understory species, were highly disturbed, and the mortality of the preceding cohort was abrupt with the concomitant increase in bottom illumination. Trickled cohorts were more common in shallower depths (≤15 m), at sites where understory was prevalent prior to cohort development, or where the mortality of the previous cohort was gradual and had not fully died off, thus leading to an overlapping or blending of cohorts (def., “Blended”—Table 3). Cohorts at the shallowest sites (≤12 m) were not well delineated into discrete pulses as those at deeper sites (e.g., PLC18; Figure 7 vs. PLC12; Figure 5). Cohort blending, in isolation of other factors, did not appear to have a significant effect on cohort longevity, but partially contributed to cohort size in some cases as shall be discussed later.

**TABLE 3:** The fraction of cohorts that appeared as trickled or pulsed, whether juvenile recruitment was observed within 3 months after at least 95% of plants in the previous cohort died, and the cause of mortality as assessed from field notes where possible (in decreasing order of importance).

Demographic event	Category	Proportion
Cohort appearance	Trickled	0.35
	Pulsed	0.65
Juvenile recruitment	Blended with previous cohort(s)	0.62
	Within 3 months after previous cohort	0.72
Mortality cause	Heat	0.25
	Not known	0.22
	Heat/wave energy	0.19
	Wave energy	0.13
	Small cohort	0.08
	Heat/sea urchins	0.06
	Sea urchins	0.03
	Wave energy/sea urchins	0.03
	Amphipod infestation	0.01

The second general characteristic was that cohort size (*N*) decreased, and cohort appearance became increasingly trickled over time, especially after the MHW of 2014–2015. A linear model of cohort size (*N*) over time was significantly negative (*F*[1,147] = 28.21, *p* ≪ 0.001). Cohorts were also more trickled over time (*p* ≪ 0.001—the binary factor of Trickled v. Pulsed for each cohort was tested using logit regression). For this analysis, cohorts were classified into either Trickled or Pulsed heuristically from the plots of species accumulation over time (second panel of Figures 4, 5, 6, 7, 8, Appendix S1: Figures S6–S20) for this analysis.

The third general characteristic was that cohort size (*N*) was positively correlated with the number of giant kelp juveniles (*r* = 0.77, *p* < 0.001) and with increasing depth (*r* = 0.20, *p* < 0.02). The rates at which juveniles survived to recruit as adults were highly variable across space and time. The only clear pattern was that shallower sites (≤15 m) appeared to have more consistent juvenile recruitment than deeper sites. Juveniles increasingly failed to recruit into adult cohorts after the 2014–2016 MHW and the winter wave events of 2021 and 2023, and in particular in the 2020s at 16 of 20 sites (Card, SB, DM, LJN12, LJN15, LJN18, LJS12, LJS15, LJS18, PLN18, PLC12, PLC15, PLC18, PLC21, PLT15, and PLT12), which was rare prior to the MHW. The reasons for the later adult recruitment failure are not known, but it was clear that plants failed to thrive.

### Factor analysis

**FIGURE 10:**
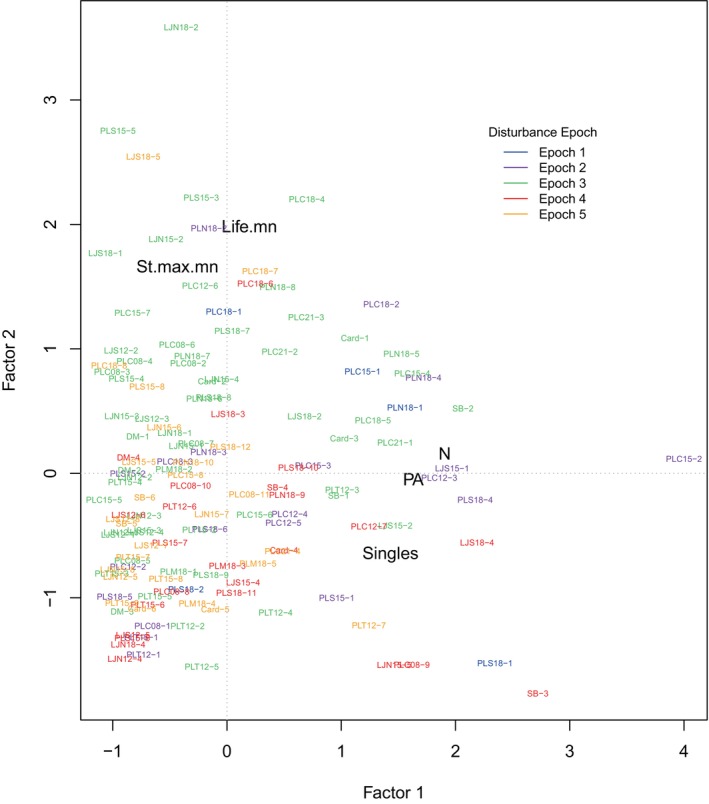
Plot of the first two factors in the factor analysis of *Macrocystis pyrifera* cohort characteristics. The first factor (43% of variance) includes characteristics related to cohort size (PA, the number of pre‐adults that developed into each cohort; singles, the number of adults observed only once in a cohort), and the number of plants in each cohort (*N*). Factor 2 (36% of variance) includes factors related to cohort longevity (Life.mn, cohort mean lifespan, St.max.mn, mean of maximum stipe number for each plant in cohort). Cohorts are identified by their site name and cohort number (e.g., PLC15‐1 indicates the first cohort at PLC15). Cohorts are color coded by disturbance epoch (see text).

The first two factors from the factor analysis (Figure 10) were related to cohort size and longevity and accounted for 43% and 36%, respectively, of overall variance (79% of total variance). Cohorts in the factor analysis plot are colored by periods between major disturbances (termed “Disturbance Epochs”) for visualization of temporal variability in cohort size and longevity during which *M. pyrifera* off San Diego County suffered large‐scale mortality. The disturbances separating the epochs include the 1982–1983 El Niño, the 200‐year storm of 1988, the 1997–1998 El Niño, the 2005–2006 El Niño, the MHW of 2014–2015, the 2018–2019 El Niño events, and the storms of the 2020s. The periods between these events were classified as Disturbance Epochs 1–5, respectively. Factor 1 included characteristics related to cohort size, including the number of adults observed only once in each cohort (Singles), the number of pre‐adults that developed into each cohort (PA), and the number of plants in each cohort (*N*). Factor 2 included factors related to cohort longevity, including mean lifespan (Life.mn) and the mean of the maximum number of stipes observed for each plant as part of each cohort (St.max.mn).

### GAM models

**FIGURE 11:**
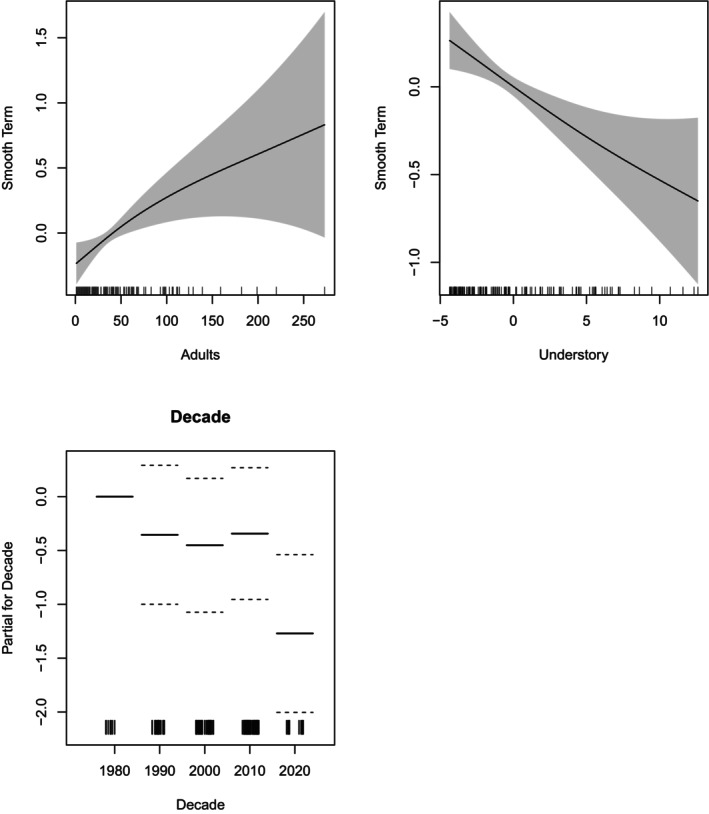
Plot of smooth terms and decade partial for the generalized additive model (GAM) analysis of cohort size (number of plants in cohort).

**FIGURE 12:**
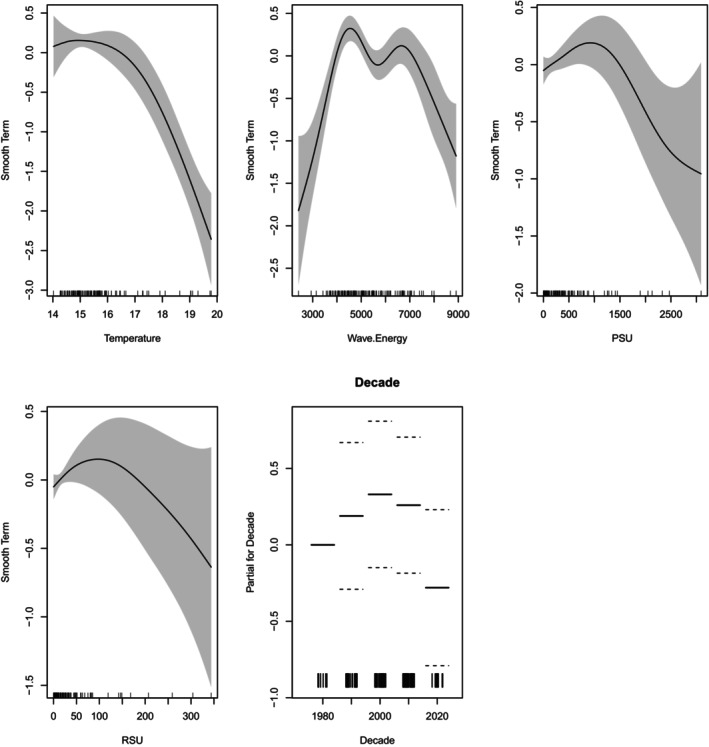
Plot of smooth terms and decade partial for the generalized additive model (GAM) analysis of cohort longevity. PSU, *S. purpuratus*; RSU, *M*. *franciscanus*.

The results of the GAM models examining cohort characteristics related to and potentially forcing the factors associated with cohort size and longevity are shown in Tables 4 and 5, and GAM smooths are shown in Figures 11 and 12, respectively. For Factor 1, cohort size (*N*) was used as the dependent variable and was significantly related to the number of adults (Ad) that existed (from a previous cohort[s]) within at most one year prior to the beginning of each cohort (emergence), and the magnitude of understory algal cover (scaled combination of all species other than *M. pyrifera*) within one year of cohort emergence (Figure 11). Cohort size increased with increasing number of previously existing adults (*p* < 0.009) likely due to the local supply of giant kelp propagules. Cohort size was negatively affected by the cover of understory algae (*p* < 0.002). Decade was included as a condition factor in the analysis and revealed that cohort size in the 2020s was significantly less than that in the 2000s (*p* < 0.0013). The median cohort size in the 2020s was less than the median values for all other decades though not significantly. Therefore, the prevalence of understory algae clearly interfered with cohort size via space competition.

**TABLE 4:** Generalized additive model (GAM) analysis table for cohort size (number of plants in a cohort).

	Estimate	SE	*z*	edf	Ref. df	χ^2^	*p*
Parametric coefficients							
(Intercept)	4.432	0.118	37.645				<2E‐016***
decade1980	0.452	0.311	1.46				0.146
decade1990	0.097	0.213	0.46				0.647
decade2010	0.108	0.154	0.70				0.483
decade2020	−0.819	0.254	−3.22				0.0013**
Significance of smooth terms							
s(Adults)				1.47	1.80	8.21	0.009**
s(Understory)				1.20	1.37	10.67	0.002**

*Note*: *R* ^2^(adj) = 0.248; deviance explained = 25.20%.

Abbreviation: edf, estimated df.

** *p* < 0.01;

*** *p* < 0.001.

**TABLE 5:** Generalized additive model (GAM) analysis table for cohort longevity.

	Estimate	SE	*z*	edf	Ref. df	χ^2^	*p*
Parametric coefficients							
(Intercept)	5.934	0.109	54.541				<2.00E‐16***
decade1980	−0.330	0.239	−1.38				0.168
decade1990	−0.141	0.170	−0.83				0.408
decade2010	−0.0704	0.157	−0.45				0.654
decade2020	−0.610	0.197	−3.11				0.002**
Significance of smooth terms							
s(Temp)				3.09	3.87	91.98	<2e‐16***
s(Waves)				5.89	7.03	57.59	<2e‐16***
s(PSU)				2.97	3.67	11.69	0.017*
s(RSU)				2.20	2.69	5.01	0.070

*Note*: *R* ^2^(adj) = 0.41; deviance explained = 59.20%.

Abbreviation: edf, estimated df; PSU, *Strongylocentrotus purpuratus*; RSU, *Mesocentrotus franciscanus*.

* *p* < 0.05;

** *p* < 0.01;

*** *p* < 0.001.

Mean lifespan was utilized as the dependent variable for the GAM indicated by Factor 2. The independent variables identified by random forest analysis and that were significant or close to significance in the Factor 2 GAM included the 80th percentile of bottom temperature at PLC15 (*p* < 0.001), the 80th percentile of significant wave energy (*p* < 0.001), and the density of red (*p* < 0.07) and purple (*p* < 0.02) sea urchins (Figure 12). The algal periods used for these independent variables were the 95th percentiles of individual cohort survival (quantile derived using Kaplan–Meier analysis). Increasing bottom temperatures were negatively related to cohort longevity; however, the relationship between wave energy and cohort longevity was more complex. Longevity was low at small wave amplitudes and increased up to a threshold sill of ~4000 Joules m^−2^ then decreased beyond ~6800 Joules m^−2^ where mass cohort mortality occurred. Increasing sea urchin densities also had a negative effect on cohort longevity at >2.5 m^−2^ for purple sea urchins and >0.25 m^−2^ for red sea urchins. The results of decade as a conditioning factor were similar to those observed for the Factor 1 GAM, with longevity in the 2020s being significantly less than in the 2000s (*p* < 0.002) and the 2020 median being less than for the remaining decades (Figure 12). Note, the relationships among the variables in both GAM analyses were robust using different quantile levels of the independent variables. The results included here were deemed most representative.

## DISCUSSION

Marine conservation and restoration have received global attention in recent decades. Knowledge of where to focus effort has been shown to be critical for successful outcomes (Fraschetti et al., 2021). Incorporating the understanding of longer term widespread demographic patterns, which, for any species, necessitates survivorship monitoring (Harper & White, 1974), has been shown to increase successful management, especially for larger and longer lived species. Unfortunately, this information is often difficult to obtain, especially in wave‐exposed, subtidal, marine ecosystems such as kelp forests.

Giant kelp, *M. pyrifera*, is one of the best‐studied marine species to date; however, surprisingly little is known regarding the mechanics of recovery after disturbance at decadal and longer time scales, and many current analyses rely on canopy presence and extent without consideration of the complex benthic patterns and processes, which are known to structure populations in space and time. This analysis of giant kelp cohorts utilizing demographic data collected over >4 decades in representative populations across different habitats off San Diego provided a rare long‐term retrospective opportunity to explore drivers of cohort recruitment, growth, and survival at a range of spatiotemporal scales. The study sites include a wide array of benthic habitats ranging from flat to very heterogeneous hard bottoms to low relief mudstones surrounded by sand, comprising stark oceanographic and seascape (Switzer et al., 2016) differences (Table 1). Because cohorts were analyzed in the context of dominant benthic algal assemblages and environmental conditions characterized over decades across different depths and oceanographic regimes (Parnell et al., 2006, 2010), this analysis provides insights into giant kelp dynamics and resilience in southern California. Also, because of the inclusion of a wide range of habitats, depths, and oceanographic climates over longer time scales, our results contribute to general models of giant kelp demography.

*M. pyrifera* is known to respond to both abiotic (such as physical forcing) and biotic (such as competition and herbivory) processes, as well as their interaction with habitat heterogeneity, warming events, and strong storms. Detected in this long‐term retrospective analysis were the most impactful physical forcing events on *M. pyrifera* in this region, including the 1983–1984, 1997–1998, and 2015–2016 El Niño events. The 2015–2016 El Niño was preceded by a marine heat wave emanating from the NE Pacific originally termed the “Blob” (Tseng et al., 2017). The net effect of these linked events was the decimation of kelp throughout southern California (Edwards, 2019; Michaud et al., 2022), the impact of which persists at most sites. The unusually large storms associated with the El Niño events of 1982–1983 and 2016 also showed particularly large impacts in our time series. Other large storms occurred in 1992, 2021, and 2023, but by far the most damaging storm was that of 1988. This storm virtually eliminated giant kelp from San Diego County and was followed by a strong La Niña, resulting in rapid recovery thereafter (Seymour et al., 1989).

Over the recent decades across the San Diego region, most of the sites that were dominated by giant kelp at the beginning of their respective time series had shifted to an understory‐dominated state by either the 2014–2016 MHW or the wave events of the early 2020s, with only two of the deeper sites classified as being in a recovery state, and no sites classified as being in a kelp‐dominated state recently. A few phase reversals occurred without prior disturbance, thus indicating that phase reversals can occur for reasons other than extreme physical disturbance, potentially due to biological forcing, but they were not common. Overall, our analysis suggests that the resilience of *M. pyrifera* has significantly decreased over the last decade at nearly all sites. Giant kelp is presently sparse throughout San Diego County. This general pattern of multidecadal decline seems to be global (Butler et al., 2020). Although biomass and adult densities of *M. pyrifera* are known to wax and wane over multiyear cycles, typically forced by ENSO and storms (Tegner & Dayton, 1987), little is known regarding the longer term survival of individual plants, especially over longer time scales.

Individual and cohort survivorship curves showed that the age structure of giant kelp stands was dominated by cohorts along a continuum of distinctiveness. Our analysis showed that the *Macrocystis* populations responded to environmental changes in three ways: (1) discrete cohort recruitment pulses, increasing in magnitude with depth; (2) phase shifts in cohorts persisting for years after major physical disturbances such as strong El Niño events and storms; and (3) spatial coherence of cohorts over time. Coherence among sites was greatest among subregions (e.g., North County, north La Jolla, south Pt. Loma), suggesting that habitat and ocean microclimate strongly modulate cohort patterns, followed by the importance of depth. The spatial coherence among subregions is highly relevant for an overall understanding of patterns in southern California, as the North County reefs are more similar to most of the other reefs in southern California with small shallow patches surrounded by sand, and thus the understory takeover in that subregion heralds what may happen to other areas in southern California.

Although cohort survival was highly variable, high mortality occurred throughout the study region within the first year of juvenile recruitment, most likely due to self‐thinning (Dayton et al., 1984; Dean et al., 1989), with a large fraction of overall mortality attributed to warming (Becheler et al., 2022), storm waves (Seymour et al., 1989), or both, as expected. Greater survivorship generally occurred at deeper sites where it is cooler and where benthic wave energy is less than at shallower sites (Seymour et al., 1989). However, this pattern of high mortality became increasingly prevalent in recent decades, accompanying a switch from kelp‐dominated benthic assemblages to understory domination. Early successional competition with *D. ligulata* delayed or lengthened the establishment of some cohorts and indirectly contributed to decreases in cohort longevity via decreased cohort population size.

This complex pattern was further shown with a larger fraction of cohorts (65%) occurring in a pulsed fashion, recruiting after major disturbance, during periods of time when competing algal stands, including understory species, were most disturbed and the mortality of the preceding cohort was abrupt, thereby releasing new recruitment from light competition during times of near complete die‐off of the previous cohort. A smaller fraction (35%) of cohorts developed gradually or in a trickled fashion, also supporting previous studies on canopy‐forming kelps posing light competition for self‐recruitment due to the light requirements for gametogenesis. A fraction of the cohorts that were of the pulsed type, typical during canopy disturbance prior to the heat wave of 2015, switched to a more trickled recruitment pattern, with increasingly reduced survivorship at shallower sites, leaving the deeper sites as essentially the only sites showing any form of recovery in the recent decade. However, these deeper sites still did not reach the giant kelp‐dominated state of decades past and are potentially the harbingers of what populations in the region will look like into the coming century if understory dominance continues.

The potential drivers of this pattern likely involve a combination of biological and physical factors. For example, at shallower sites, sufficient light for gametogenesis occurs more frequently (Deysher & Dean, 1984, 1986), which is an important step in recruitment, but the drawback is the co‐occurrence of increased warming and wave action in the shallows, which are known sources of mortality for kelp recruits, supported by this study and other studies (see above). However, and importantly, cohort recruitment, which is absolutely necessary for post‐disturbance recovery and for population resilience, has become increasingly limited by competition with understory (Dayton et al., 1992, 1999), potentially due to reduced benthic light or space (Dean et al., 1989), even under cool conditions, conducive for giant kelp. This has resulted in many sites experiencing persistent recruitment failure and overall reduced resilience across our study region.

Competition for space is a well‐known factor limiting recruitment and the successful establishment of the initial stages of giant kelp. The pattern of understory guilds thriving in the absence of surface canopy has been shown in other regions, where a reduction in both adult kelp density and recruitment has occurred due to an increase in understory macroalgal assemblages such as turf in a sub‐Antarctic kelp forest (Friedlander et al., 2020; Kaminsky et al., 2024). Chemical suppression of kelp gametophytes by red turf algae in the Gulf of Maine has recently been reported (Farrell et al., 2025), representing a novel mechanism of space competition between turf algae and kelp species, thus potentially reducing kelp resilience. Understory guilds comprised of stipitate and fleshy kelps as well as brown and red turf compete for both space and light during giant kelp recruitment (Dayton, 1975). Drifting spores need to settle to available space on hard substrata (Charters et al., 1973), produce gametophytes, and develop and release gametes controlled by adequate light (Neushul, 1963), and then grow into juvenile blade‐stage plants without being grazed or removed by water motion or sand scour (Devinny & Volse, 1978). The competition for space and light at this critical stage in the kelp life history likely explains why cohort initiation in our longer term data was inversely related to understory and might also explain why cohort recruitment was mostly pulsed after disturbance. For example, storm waves and warming would obviously damage giant kelp, but also remove understory, thereby releasing giant kelp recruitment from competition for space and light with both understory and self‐shading canopies. However, the increasingly trickled nature of recent recruitment events, rather than the pulsed events of decades past, could be due to increased understory competition for space during and after disturbance. Trickled cohorts were more common shallower at sites where understory was prevalent prior to cohort development, or where the mortality of the previous cohort was gradual rather than abrupt. And trickled cohorts became more prevalent over time, further supporting the widespread increase in understory competition posing a challenge to large giant kelp recruitment pulses.

The age distribution of giant kelp was less skewed at sites that experienced more continuous and trickled recruitment (i.e., cohort blending). This was observed more frequently at shallower sites where light can support giant kelp recruitment even in the proximity of established canopy. When cohorts recruit in a blended overlapping fashion, rather than pulsed, the demography of the population age structure becomes staggered. The importance of mixed aged structure on the population dynamics of marine plants is poorly understood, but is known to vary among terrestrial systems (Coomes & Allen, 2007) with a clear hierarchy of dominance and suppression of understory via competition for light (Mohler et al., 1978). In contrast, shallow coastal marine environments are more characterized by mechanical disturbances that might partially mitigate competitive dominance.

Biological factors influencing kelp populations often involve several guilds of herbivores (see Edwards, 2022). In our study region, these include small micrograzers that are most abundant in geniculate coralline turf algae. This guild, composed mostly of small crustacea and polychaetes, grazes on and can eliminate small kelp sporophytes (Dayton et al., 1984). Other small grazers include damaging benthic amphipods (Gutow et al., 2020). Populations of these benthic crustacea respond when their small fish predators are reduced or eliminated in response to strong El Niño events (Tegner & Dayton, 1987). As their principal predators disappear, these amphipods can colonize kelp fronds and consume them just as the canopies recover from the typical nutrient starvation of an El Niño or heat wave event. These and other canopy fish consume invertebrates that settle as epiphytes on kelp fronds, especially on the outer edges of kelp forests where fish incidentally consume the kelps (Bernstein & Jung, 1979). In our study, amphipods exhibited a significant effect on cohort truncation for a small fraction of cohorts, potentially related to greater understory presence. The combined effects of competition for space and light by understory, as described above, combined with the increased presence of micrograzers in understory guilds as a persistent response to dwindling surface canopies during warm, low nutrient events, could certainly be structuring these populations over time by preventing recruitment and could explain the pattern of decreased cohort initiation after disturbance where and when understory dominated.

Sea urchins are also well known to be destructive grazers of kelps at mid‐latitudes (Dayton, 1985; Mann, 1977; Vadas, 1977) and have been implicated in massive die‐offs in California (Rogers‐Bennett & Catton, 2019). In our retrospective study, purple sea urchins showed a significant effect on cohort mortality in the GAM analysis, principally driven by their impact in the southern Pt. Loma sites in proximity to a resilient sea urchin barren (Parnell, 2015). Sea urchins were particularly relevant at this one deeper site where they modified the general pattern of increased longevity and survivorship overall at depth but were not a common factor of mortality across all sites. The San Diego region appears to be an exception to the general pattern of increased sea urchin grazing as a primary driver of community structure (Ling & Johnson, 2009; Parnell et al., 2017).

Greater cohort survivorship occurred at deeper sites with reduced sea urchin influence. The pattern of greater survivorship found at depth in this study was expected due to the beneficial colder conditions for survival and growth in deeper water, where hard substrate exists, particularly in the persistent Pt. Loma giant kelp forests, and because deeper areas are less susceptible to damaging wave energy, although these environments do pose a light challenge for recruitment. The decadal pattern of increasing survivorship at depth relative to shallower sites is probably due to shallow warming due to climate change, combined with reduced recruitment due to increased understory presence. This complex spatiotemporal pattern would not have been tractable had it not been for longer term permanent transect monitoring. Going forward, the persistent recruitment failure at shallow, light‐replete sites may only be compensated for by occasional trickle recruitment in deeper, colder waters, and only when sufficient light is available at times when understory guilds are not dominant on the deeper benthos.

In general, kelp is known to show greater survivorship at depth due to the combined effects of reduced wave action and cooler water during disturbance (Ladah & Zertuche‐Gonzalez, 2004; Seymour et al., 1989), although many sites in this region do not have hard substrate deeper than 20 m (Emery, 1960). Our data showed a pattern of increasing differences between depths over time, potentially due to permanent disturbance at shallow sites after severe MHWs and recent storms. For example, in our study, cohort size significantly decreased over time, with fewer pulsed recruitment events, and this trend was positively related to the existing number of giant kelp juveniles and with increasing depth, and negatively with the magnitude of understory algal cover within one year, which has increased at most of our sites. Although shallower sites showed more consistent juvenile recruitment than deeper sites, likely due to an increased light effect, they also exhibited lower survivorship due to many of the factors discussed above. Juveniles increasingly failed to mature to adulthood after the 2014–2016 MHW and the winter wave events of 2021 and 2023, with adult recruitment failure in the 2020s at most sites, which was rare prior to 2015. Competition with understory and recruitment failure leaves the deeper sites as the only sites with relatively abundant giant kelp even in this recent cooler environment. Even those sites, however, remain in a recovery pattern rather than reverting to a kelp‐dominated state as had typically been the case prior to the MHW of 2015.

For most cohorts, juvenile recruitment occurred within 3 months of the previous cohort's die‐off for pulsed recruitment events. This was due to conspecific light competition beneath the giant kelp canopy as shown in previous studies, but the occurrence of pulsed events has decreased over time. Cohort longevity was also inversely related to bottom temperature and has significantly reduced over time as well. The relationship between wave energy and cohort longevity was complex, with longevity being low at both small and large wave amplitudes. Wave action is necessary for water‐column mixing and for nutrient uptake at blades to avoid a boundary layer and to avoid disease, yet high wave action can damage both adults and juvenile giant kelp as well as recruits, particularly in areas with low relief or unstable substrate.

The demographic data available from this study are useful for comparison with data from previous studies to gauge how the size distributions of *M. pyrifera* have changed over multidecadal time scales. For example, plant size was highly correlated with longevity (e.g., Figure 10). Such comparisons demonstrate that plant sizes off San Diego have decreased substantially since 1976. The mean of the maximum number of stipes for each cohort off Del Mar in this study was at most half of that observed for the 1967–1970 period. Rosenthal et al. (1974) estimated this mean as 49.8 in the late 1960s, whereas the means for the four cohorts we observed off Del Mar ranged from 11.3 to 27.8 (Appendix S1: Table S1). There has been no giant kelp off Del Mar since late 2015 (Appendix S1: Figure S8). Comparisons of the same metric at the PLC15 site indicate that the median number of stipes observed for each plant in 1957 and 1974 were ~60 and ~40, respectively (Tegner et al., 1996—their fig. 10). The same values from this study for the PLC15 site for cohorts 1–8 were 21, 12, 15.5, 18, 37, 8, 32.5, and 16.5, respectively. Only two cohorts from this study approached the plant size distributions from 1974. And no new cohorts appeared at this site after the 2016 cohort (#8) with complete mortality by 2020.

In general, the well‐known patterns of abiotic controls on kelp survival related to thermal (most likely nutrient‐related) and storm wave disturbances were found in this long‐term study. The impact of increasing competition over time with understory at shallower sites, and increased survivorship at deeper sites, emphasizes the complex interplay between (1) competition for space and light both with self‐shading surface canopy kelps and with all forms of understory species including turf, (2) survival and recruitment during extreme events, and (3) the need for the nearby presence of reproductive adults for recruitment post disturbance (Dayton et al., 1984; Reed et al., 2024). The patterns observed over the decades indicate a trend of more trickled cohorts rather than pulsed ones. There has been an increase in understory recruitment at shallower locations, while survival rates of giant kelp are greatest at deeper sites devoid of sea urchins. This implies that the kelp populations in the shallower waters off San Diego County will be more transient and could require reseeding from the deeper, long‐lived populations that are less exposed to wave action. These deeper populations, which thrive on hard substrates in the cooler, darker waters off Point Loma, might be essential for sustaining regional kelp in the future.

A primary consideration for marine ecosystem restoration is spatial prioritization (Fraschetti et al., 2021). Our analysis suggests that depth is one of the greatest modulators of cohort survivorship and recruitment dynamics and suggests that a focus on deeper reefs for both restoration and conservation efforts is warranted. Although there exists a general ecological understanding that strongly interacting species are an important part of ecosystem function, understanding how different assemblages modify cohort survivorship and recruitment dynamics over longer term processes spanning many decades is hard to come by in the marine literature. Yet, it is needed to design effective conservation measures (Hillebrand et al., 2020). Understanding species turnover and especially species decadal dominance shifts is particularly important for marine conservation efforts (Hillebrand et al., 2020). The quantification of the temporal and spatial change of an ecosystem and how such changes affect the processes characterizing marine ecosystems are critical. Gårdmark and Huss (2020) show how responses to stressors can alter individual performance, population size structure, and finally, food web dynamics, and the need to consider these responses in the development and application of conservation efforts. Additionally, cohort and demographic analysis of plants over longer time periods is crucial for designing and modeling conservation outcomes (Schwartz & Brigham, 2003).

Another important consideration for remediation planning is the spacing of resilient patches of giant kelp for the provision of spores to nearby areas. Patches of giant kelp have been observed to be capable of seeding areas as distant as a few kilometers (Reed et al., 2024). Cohort development in our study prior to the MHW appeared generally coherent among study site subregions (areas separated by 5–10 km). However, this pattern appeared to break down after the MHW as understory domination increased, and the proportion of cohorts whose recruitment was trickled at shallower depths increased over time. This indicates the strong resilience of understory canopy to competitively exclude giant kelp in shallower areas even when nearby spore sources are available. This suggests that giant kelp remediation efforts will likely fail over time scales longer than a cohort generational period without repeated episodes of understory clearing and the maintenance of nearby spore sources.

The failure of giant kelp resilience after the MHW was most pronounced at the North County and northern La Jolla sites where the understory has become dominant. This contrasts with the cause of giant kelp decline in most other areas off California where overgrazing by sea urchins has been most problematic. Sea urchin grazing affected cohort longevity in this study, but not giant kelp resilience. This raises the question of whether the lack of giant kelp canopy in areas due to understory domination warrants remediation as understory canopy represents a “natural” state and is likely to increase over time with continued warming. This suggests that a value judgment comes into focus of whether remediation should include the removal of understory canopies for giant kelp recovery, and, if so, in which cases.

What is clear is that the kelp forests off San Diego County are now profoundly different from the robust forests of the 1970s and the stands present at the beginning of this time series. Large synchronized cohort pulsing is now greatly diminished and the age structure of giant kelp has become skewed younger and smaller as understory has encroached into areas once dominated by surface canopies. The increasingly trickled nature of giant kelp cohort recruitment heralds a new era of giant kelp forest dynamics with profound implications for conservation efforts.

## AUTHOR CONTRIBUTIONS

P. Edward Parnell, Paul K. Dayton, and Lydia B. Ladah contributed to the conceptualization, writing, and editing of this manuscript. Analyses were conducted by P. Edward Parnell and Cleridy E. Lennert‐Cody. Figures were created by P. Edward Parnell. The remaining authors assisted with writing, editing, and discussions regarding the scope and content.

## CONFLICT OF INTEREST STATEMENT

The authors declare no conflicts of interest.

## Supporting information


Appendix S1.

## Data Availability

Data (Parnell et al., 2025) are available in Dryad at https://doi.org/10.5061/dryad.fttdz096d.
