# Supplementary material for: Demography and dynamics of giant kelp cohorts across four decades: Lessons for conservation and resilience planning
Source: Ecol Appl. 2026 Jan 28;36(1):e70181. doi: 10.1002/eap.70181 (PMC12851851; doi:10.1002/eap.70181)
Supplement: Supplementary file 1 — Appendix S1. [file EAP-36-e70181-s001.pdf]

Appendix S1

Demography and dynamics of Giant Kelp cohorts across four decades: Lessons for conservation and resilience planning

P. Edward Parnell, Cleridy E. Lennert-Cody, Lydia. B. Ladah, Kristin L. Riser, Brenna Bulach, James J. Leichter, Ami Latker, Stephen C. Schroeter, Paul K. Dayton

Ecological Applications

Equation (S1)

$$P = 0.5 * \rho * g * H^2 \quad * T$$

P = wave power

ρ = density of seawater (1000 kg m^{-3})

g = gravitational acceleration (9.81 m s^{-2})

H = wave height

T = wave period

Table S1. Cohort parameters used in GAM models. 'RSU'=Red Sea Urchins, 'PSU'=Purple Sea Urchins. Wave energy units are J m_{-2}. Understory values are scaled among all cohorts. T=temperature (°C). Lifespan is in days. Q95 is Kaplan-Meier derived 95th percentile of lifespan.

Site	Cohort	N	Existing Adults	T0	Mean Max Stipes	Q95 (d)	Mean Lifespan (d)	Understory	T	Wave Energy	RSU	PSU
Card	1	206	97	2006-12-06	22.3	1540	692	-3.52	14.8	6969	51	62
Card	2	74	15	2011-08-23	25.7	1399	576	-3.96	15.7	4570	259	1197
Card	3	183	93	2012-08-16	20.5	1119	490	-3.34	16.3	4005	149	54
Card	4	145	61	2016-08-30	8.3	644	257	-3.54	15.2	6192	5	17
Card	5	74	107	2017-11-07	7.1	288	125	-3.48	15.5	4011	8	30
Card	6	27	4	2020-08-24	6.2	288	112	-0.30	14.9	4694	0	0
SB	1	182	10	2006-10-20	20.6	930	374	-3.00	14.5	7019	2	0
SB	2	234	4	2010-09-03	23.7	1445	573	-2.79	15.0	4775	0	14
SB	3	239	64	2014-10-31	5.6	203	28	-4.31	17.4	4494	0	2
SB	4	145	32	2016-08-30	12.6	644	380	-1.11	15.2	6192	0	0
SB	5	15	3	2019-08-28	12.3	917	278	-1.41	15.2	4481	0	0
SB	6	41	19	2020-08-24	23.6	744	329	0.76	14.9	4715	0	0
DM	1	25	7	2007-01-29	16.4	1280	454	-1.31	14.8	6907	0	0
DM	2	21	18	2008-05-20	18.2	823	383	-1.09	15.0	7486	0	0
DM	3	14	20	2010-05-19	11.3	391	100	-0.99	14.0	5793	0	1
DM	4	29	14	2013-02-27	27.8	835	404	-0.54	16.1	3841	0	0
LJN12	1	20	16	2004-06-30	22.6	714	254	3.41	14.9	5064	8	117
LJN12	2	32	8	2006-12-08	26.3	749	366	4.04	14.5	7044	5	6
LJN12	3	41	15	2013-02-26	23.5	618	292	6.25	15.5	3560	19	18
LJN12	4	12	10	2015-08-27	4.7	98	8	6.63	19.8	5821	10	9
LJN12	5	19	2	2017-08-31	19.4	376	166	8.61	16.0	3751	9	7
LJN15	1	73	45	2004-11-30	20.8	624	442	2.57	14.8	5276	50	148
LJN15	2	53	53	2007-02-21	31.3	1630	850	1.87	14.7	6717	50	380
LJN15	3	15	59	2010-05-05	20.9	1288	489	1.50	14.6	5499	48	325
LJN15	4	101	34	2012-03-05	28.8	994	580	1.83	15.6	4330	50	250
LJN15	5	188	59	2015-02-26	5.7	203	54	1.50	16.5	2937	50	250
LJN15	6	46	182	2016-05-19	16.9	1356	477	5.60	15.7	5612	29	83

LJN15	7	89	44	2017-08-31	17.4	729	311	9.46	16.0	5439	33	110
LJN15	8	14	83	2019-01-30	17.6	498	181	8.28	15.4	5318	32	215
LJN18	1	74	68	2004-06-11	29.3	727	462	-2.73	14.9	5036	63	66
LJN18	2	62	1	2007-08-30	38.1	2251	1277	3.20	14.8	6401	32	88
LJN18	3	9	40	2012-08-23	44.7	1197	964	NA	NA	NA	NA	NA
LJN18	4	12	20	2015-08-11	4.5	209	38	5.48	19.1	7998	47	89
LJN18	5	6	1	2019-01-29	11.0	498	255	NA	NA	NA	NA	NA
LJS12	1	27	24	2004-07-09	34.8	654	244	2.76	14.9	5353	16	44
LJS12	2	26	22	2007-03-07	36.8	986	615	4.46	14.7	6054	5	49
LJS12	3	47	15	2009-11-17	33.9	1266	485	4.29	14.9	6723	4	63
LJS12	4	43	25	2013-02-28	22.8	617	260	5.20	15.5	3560	2	48
LJS12	5	20	23	2015-06-11	9.4	221	56	5.57	19.0	6087	5	23
LJS12	6	22	22	2016-02-08	22.6	712	291	6.07	15.4	6052	1	8
LJS12	7	46	14	2017-06-06	21.2	456	235	12.35	15.8	3607	0	35
LJS12	8	21	6	2019-09-05	20.9	902	282	11.59	15.2	4461	2	11
LJS15	1	290	273	1994-01-28	18.6	1306	451	-3.64	15.0	5175	1	88
LJS15	2	229	139	1998-11-11	16.0	1104	327	-0.62	14.3	4183	7	0
LJS15	3	27	22	2002-11-05	13.2	1091	267	0.91	14.9	4824	10	3
LJS15	4	97	15	2015-08-25	13.9	714	183	2.74	16.3	7905	2	8
LJS15	5	37	59	2017-08-28	30.1	1013	397	4.61	15.9	5090	0	13
LJS18	1	21	21	2004-03-17	52.1	881	801	-3.93	14.9	4500	29	49
LJS18	2	148	15	2007-03-07	22.7	2019	526	-2.22	14.7	6675	21	15
LJS18	3	98	24	2012-08-08	31.4	1098	510	-3.39	16.1	4080	37	74
LJS18	4	261	31	2016-08-25	11.3	1519	311	-1.51	15.6	5432	11	30
LJS18	5	35	98	2018-03-13	38.5	1694	1009	0.88	15.4	4743	17	31
PLN18	1	229	159	1984-08-29	19.1	1255	564	-2.04	15.8	4130	63	363
PLN18	2	92	37	1988-09-20	32.5	1609	880	-4.27	15.0	4310	15	778
PLN18	3	82	129	1990-01-28	13.3	1018	438	-3.76	15.0	4668	15	778
PLN18	4	246	41	1993-10-06	22.2	1501	626	-3.76	15.5	5025	0	563
PLN18	5	246	13	1998-10-22	18.8	2023	671	-2.40	14.4	4696	21	678
PLN18	6	80	40	2004-08-04	27.6	1576	538	-4.05	14.8	5515	14	581
PLN18	7	55	63	2006-11-08	26.8	3187	622	-3.38	15.2	5836	25	678
PLN18	8	142	10	2010-11-30	30.9	1570	776	-2.80	15.5	5172	27	667

PLN18	9	143	22	2016-05-17	11.2	1073	367	-2.08	15.7	5983	12	304
PLN18	10	65	112	2017-11-08	13.9	809	413	-1.38	15.9	5488	8	237
PLC08	1	46	44	1997-06-24	17.7	176	74	3.14	19.3	3715	29	284
PLC08	2	77	9	1998-06-04	30.1	1246	605	3.27	14.6	3925	44	313
PLC08	3	14	62	2000-12-07	31.9	1525	572	4.33	14.7	5004	35	193
PLC08	4	24	9	2005-08-09	25.9	1297	600	5.63	14.5	6214	44	288
PLC08	5	25	12	2007-08-22	20.3	970	201	5.41	15.0	7178	46	317
PLC08	6	64	19	2009-08-12	31.3	1430	640	6.41	15.0	6483	25	510
PLC08	7	83	40	2012-08-15	27.6	897	446	7.18	16.3	4218	7	268
PLC08	8	55	96	2014-09-04	10.9	401	150	7.21	18.1	3732	7	268
PLC08	9	185	114	2015-06-10	5.5	220	58	6.75	19.0	6087	7	268
PLC08	10	69	199	2016-05-05	18.0	798	365	10.73	15.3	5179	1	101
PLC08	11	124	56	2017-08-03	19.8	933	358	12.62	16.0	5324	1	154
PLC08	12	14	16	2021-02-24	31.2	NA	383	NA	NA	NA	NA	NA
PLC08	13	22	19	2023-03-28	11.7	NA	69	NA	NA	NA	NA	NA
PLC12	1	346	442	1984-05-23	13.3	1207	404	NA	NA	NA	NA	NA
PLC12	2	22	10	1988-09-21	16.4	764	189	-3.02	14.7	3915	1	168
PLC12	3	241	103	1989-07-06	12.7	1435	434	-1.90	15.0	4660	0	65
PLC12	4	143	37	1993-06-10	15.2	1104	328	-3.19	15.2	5051	8	232
PLC12	5	148	54	1995-10-06	19.0	703	308	-2.79	15.3	4745	5	327
PLC12	6	84	99	1998-06-04	31.3	1616	763	-0.36	14.5	4206	0	154
PLC12	7	204	11	2015-04-29	11.6	725	322	4.62	16.6	7560	0	26
PLC12	8	13	10	2019-08-13	39.8	1321	658	NA	NA	NA	NA	NA
PLC15	1	215	220	1985-01-24	25.1	1441	625	-1.21	15.1	4069	1	676
PLC15	2	441	77	1988-05-27	15.6	1822	520	-2.40	15.0	4285	0	264
PLC15	3	150	107	1994-05-19	23.3	1289	428	-0.33	15.6	5126	1	512
PLC15	4	233	34	1998-09-25	21.0	1995	634	2.45	14.5	4748	5	382
PLC15	5	16	39	2004-05-21	34.8	1386	311	-1.40	14.8	5534	8	368
PLC15	6	111	5	2006-08-09	16.3	1103	320	1.25	14.5	6560	5	229
PLC15	7	32	8	2011-08-19	37.2	1383	692	1.99	15.7	4603	0	351
PLC15	8	66	6	2016-05-04	23.5	1342	383	2.58	15.7	5606	1	147
PLC18	1	97	20	1984-08-29	26.4	2068	718	2.34	15.2	4050	81	1284
PLC18	2	208	40	1988-09-21	25.6	2047	765	-0.25	15.0	4454	38	1449

PLC18	3	56	67	1995-02-21	19.2	915	409	-0.95	14.9	5014	75	888
PLC18	4	150	58	1999-01-13	29.6	2680	959	7.29	14.5	4781	24	796
PLC18	5	201	111	2006-11-02	20.5	2193	532	-1.82	14.8	6703	80	984
PLC18	6	114	62	2012-08-13	29.7	1598	779	-2.84	16.4	5456	119	1256
PLC18	7	124	37	2017-03-09	30.5	1885	805	4.50	15.4	5098	59	678
PLC18	8	16	107	2019-02-28	37.9	1622	581	1.85	15.1	4893	68	550
PLC21	1	239	49	1999-09-28	20.5	2313	491	-0.82	14.6	4894	20	200
PLC21	2	144	12	2007-05-01	24.0	1992	649	-3.07	14.8	6618	14	792
PLC21	3	149	28	2011-11-02	26.2	1412	723	-3.66	16.0	4345	26	862
PLC21	4	127	13	2018-11-02	14.6	541	252	0.83	15.7	6136	14	102
PLS15	1	176	124	1994-01-24	13.1	394	170	-3.70	15.3	5328	0	17
PLS15	2	24	42	1996-11-05	23.8	589	374	-1.92	17.3	6692	1	47
PLS15	3	88	56	1999-10-23	35.9	2169	939	-0.58	14.6	4824	1	67
PLS15	4	24	23	2005-04-27	28.8	1283	562	-2.26	14.3	5030	1	76
PLS15	5	16	3	2011-08-16	41.2	1401	1055	-0.54	15.7	4538	1	30
PLS15	6	18	15	2015-02-24	6.0	295	51	-1.20	18.6	5101	1	30
PLS15	7	49	28	2016-08-23	10.8	454	248	-0.48	15.0	6626	0	23
PLS15	8	40	42	2017-11-20	29.4	1190	550	1.16	15.6	5219	0	90
PLS15	9	13	7	2021-12-08	17.5	NA	223	NA	NA	NA	NA	NA
PLS18	1	244	47	1984-07-06	4.9	266	77	-4.24	16.7	3944	51	228
PLS18	2	61	41	1986-09-30	9.3	504	159	-4.03	15.7	4613	83	419
PLS18	3	11	54	1987-07-02	15.5	68	12	NA	NA	NA	NA	NA
PLS18	4	231	33	1992-01-15	15.5	953	395	-3.98	15.3	4243	9	0
PLS18	5	17	106	1994-06-06	18.9	381	125	-4.32	15.5	6143	0	0
PLS18	6	75	32	1996-07-10	16.1	700	282	-3.46	17.1	5893	1	6
PLS18	7	103	7	1999-07-22	28.1	1666	681	0.22	14.5	4713	3	218
PLS18	8	82	19	2006-12-05	24.6	1073	544	-4.36	14.5	6749	31	494
PLS18	9	64	6	2010-09-08	9.8	1193	193	-3.03	14.9	5630	142	2049
PLS18	10	150	107	2013-02-14	21.8	1035	421	-3.60	17.3	4055	60	276
PLS18	11	96	33	2016-08-23	9.1	289	162	-3.62	14.8	8907	3	2
PLS18	12	101	19	2018-05-30	23.8	1167	448	-2.96	15.6	4692	2	6
PLS18	13	58	4	2023-09-22	4.4	NA	5	NA	NA	NA	NA	NA
PLT12	1	23	33	1997-10-20	5.0	79	21	-2.68	19.7	5505	22	2131

PLT12	2	48	12	1998-10-12	7.0	366	87	-2.30	14.3	3682	344	1409
PLT12	3	187	44	2006-12-04	18.8	1008	386	-3.86	14.4	6791	37	392
PLT12	4	128	75	2010-09-08	11.6	534	130	0.16	14.3	5474	304	3090
PLT12	5	50	46	2013-11-07	4.9	91	9	-4.16	15.7	3144	207	2331
PLT12	6	56	3	2016-05-11	9.4	812	324	-3.53	15.3	4948	3	196
PLT12	7	191	49	2017-08-29	8.6	357	124	-2.99	15.9	3816	12	392
PLT12	8	8	17	2019-08-21	5.4	379	119	NA	NA	NA	NA	NA
PLT15	1	26	16	1997-06-16	7.0	126	54	-1.85	17.5	2414	0	435
PLT15	2	75	33	1999-03-10	15.8	643	278	-1.23	14.3	3423	6	1338
PLT15	3	13	69	2000-06-16	19.5	360	171	0.59	14.6	4092	19	2468
PLT15	4	21	11	2007-11-15	22.7	561	356	-4.16	14.5	7399	85	1254
PLT15	5	41	25	2008-08-19	12.2	326	136	-3.92	14.8	8675	85	1254
PLT15	6	31	10	2016-08-23	6.2	281	121	-4.00	14.8	8910	3	612
PLT15	7	20	10	2019-08-21	10.4	833	212	-3.17	15.2	4216	0	737
PLT15	8	43	21	2020-09-03	13.4	638	174	-2.20	15.0	5516	6	542
PLT15	9	13	35	2021-08-13	10.9	458	117	-1.33	15.1	5531	6	542
PLT15	10	22	15	2022-06-03	5.5	312	70	NA	NA	NA	NA	NA
PLM18	1	57	42	1999-10-13	15.2	524	190	-0.25	14.4	5114	19	712
PLM18	2	59	4	2006-12-04	24.7	832	391	-4.08	14.5	7073	168	1898
PLM18	3	85	1	2014-10-30	10.2	637	214	-4.26	17.9	5723	146	483
PLM18	4	69	28	2019-08-21	7.6	379	134	-3.77	15.5	4430	9	409
PLM18	5	99	34	2020-09-03	9.1	543	225	-2.67	15.0	4402	33	311
PLM18	6	12	65	2022-08-17	6.8	191	28	NA	NA	NA	NA	NA

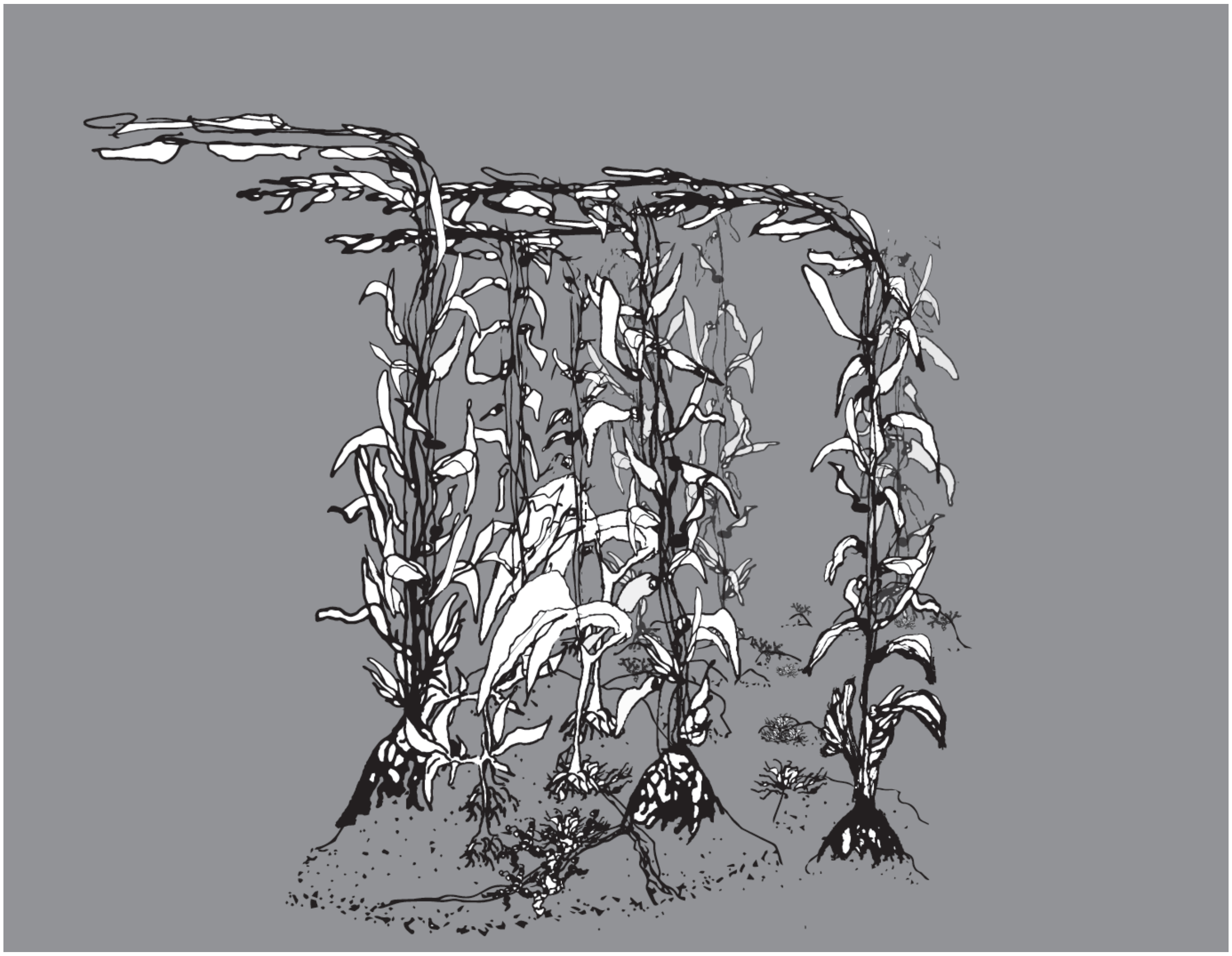

Figure S1. Illustration depicting algal state represented by Cluster 1 (‘Kelp’ state) color coded as green in Figs. 4-8 and Figs. S6-S20. Illustration created by Carlos Hernandez (source: Flickr, https://www.flickr.com/photos/203137994@N05/54658864586/in/dateposted-public/; license: CC BY 4.0, https://creativecommons.org/licenses/by/4.0/) and modified for this publication.

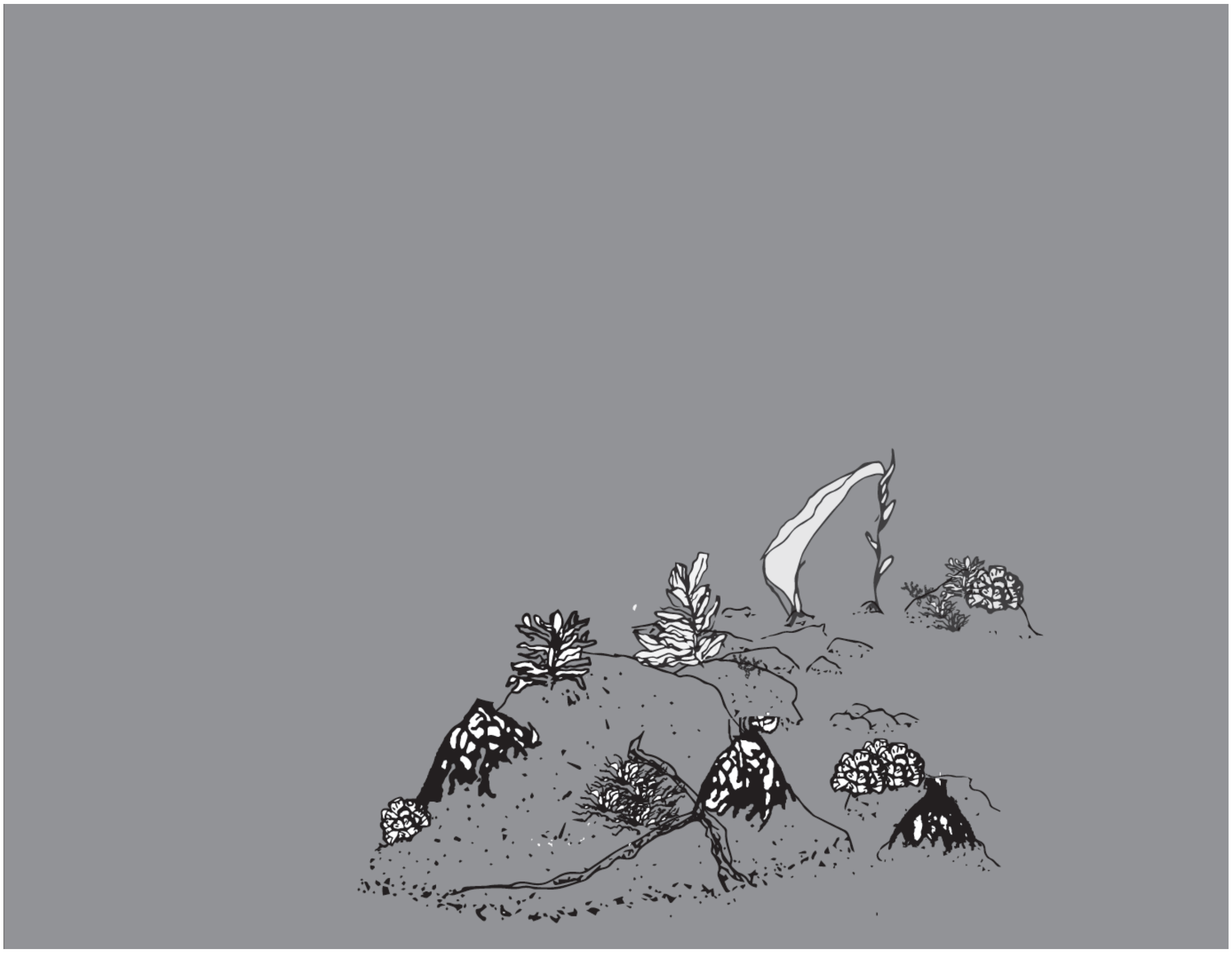

Figure S2. Illustration depicting algal state represented by Cluster 2 (‘Disturbed’ state) color coded as red in top panels of Figs. 4-8 and Figs. S6-S20. Illustration created by Carlos Hernandez (source: Flickr, https://www.flickr.com/photos/203137994@N05/54658864586/in/dateposted-public/; license: CC BY 4.0, https://creativecommons.org/licenses/by/4.0/) and modified for this publication.

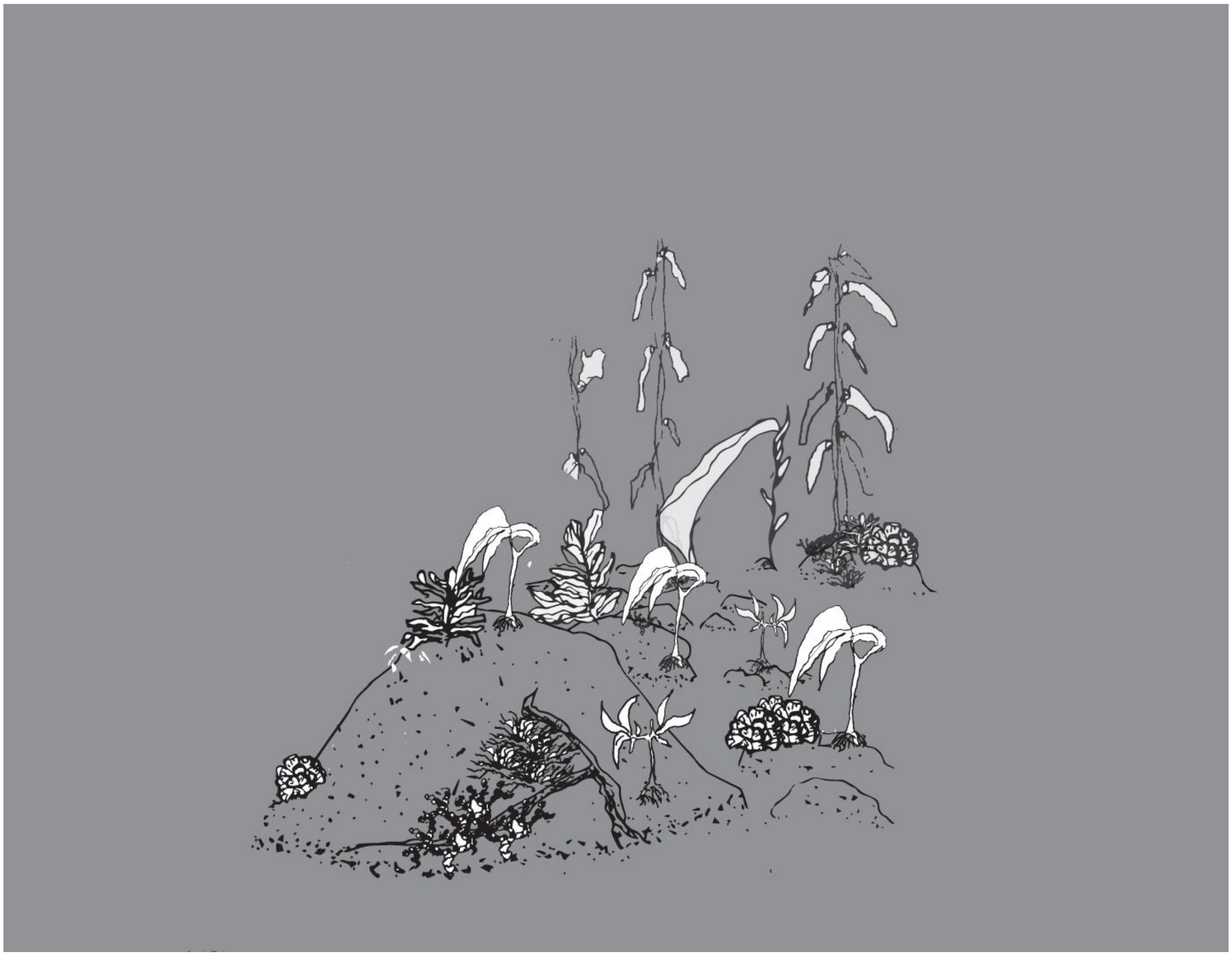

Figure S3. Illustration depicting algal state represented by Cluster 3 (‘Recovery’ state) color coded as blue in top panels of Figs. 4-8 and Figs. S6-S20. Illustration created by Carlos Hernandez (source: Flickr, https://www.flickr.com/photos/203137994@N05/54658864586/in/dateposted-public/; license: CC BY 4.0, https://creativecommons.org/licenses/by/4.0/) and modified for this publication.

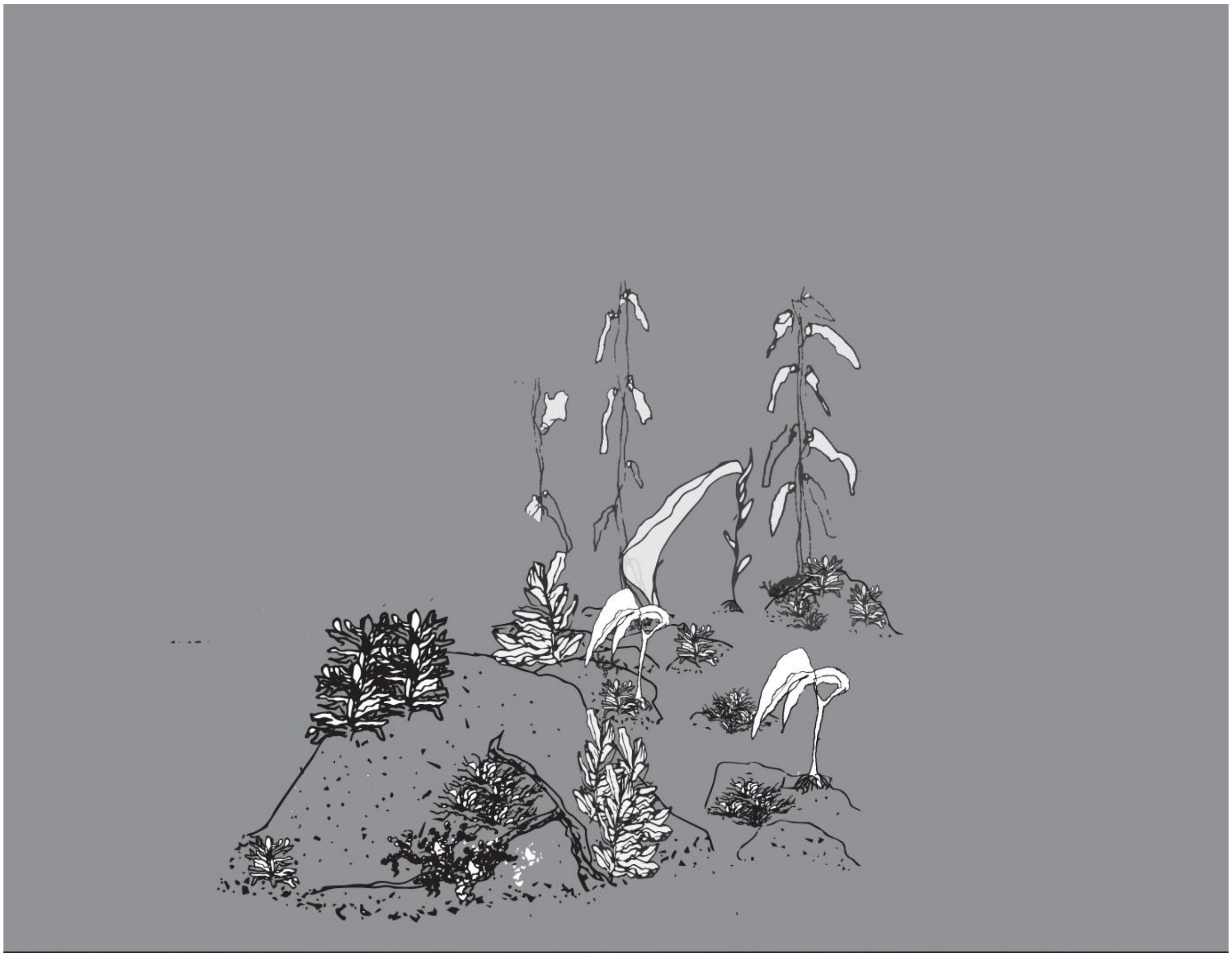

Figure S4. Illustration depicting algal state represented by Cluster 4 (‘Mixed’ state) color coded as aqua in top panels of Figs. 4-8 and Figs. S6-S20. Illustration created by Carlos Hernandez (source: Flickr, https://www.flickr.com/photos/203137994@N05/54658864586/in/dateposted-public/; license: CC BY 4.0, https://creativecommons.org/licenses/by/4.0/) and modified for this publication.

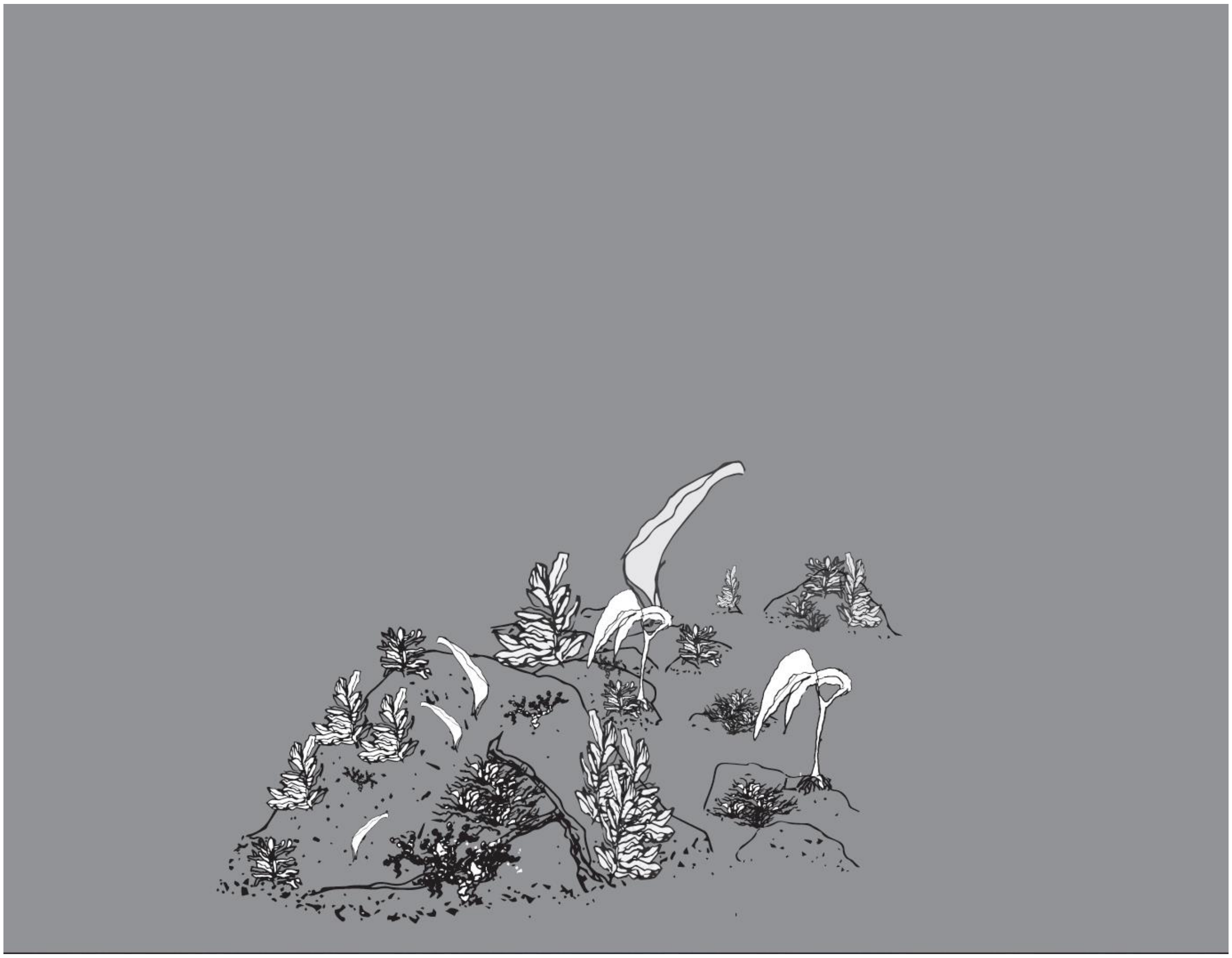

Figure S5. Illustration depicting algal state represented by Cluster 5 (‘Understory’ state) color coded as orange in top panels of Figs. 4-8 and Figs. S6-S20. Illustration created by Carlos Hernandez (source: Flickr, https://www.flickr.com/photos/203137994@N05/54658864586/in/dateposted-public/; license: CC BY 4.0, https://creativecommons.org/licenses/by/4.0/) and modified for this publication.

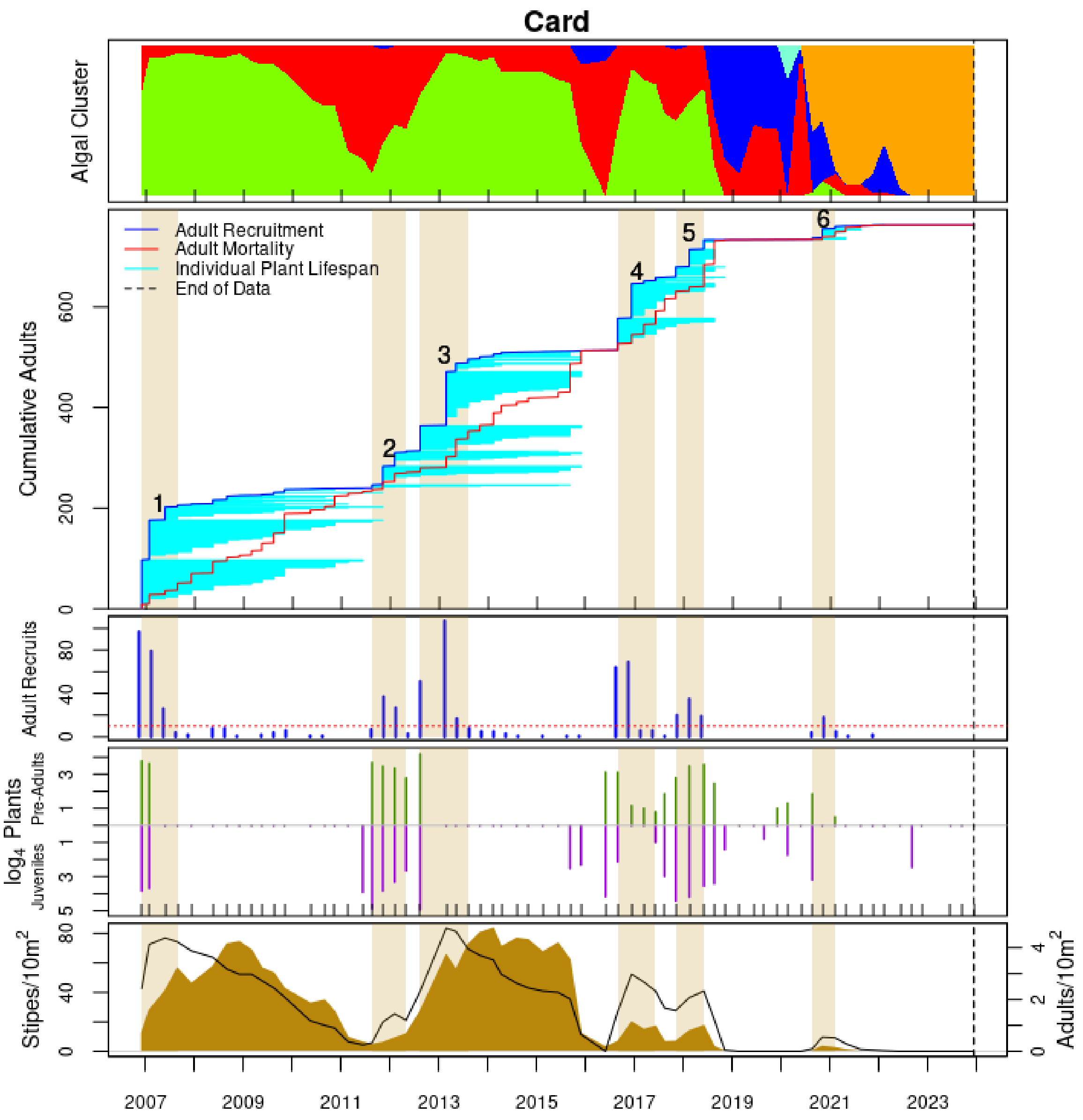

Figure S6. Timelines of algal cluster composition (top panel) – green='Kelp', red='Disturbed', blue='Recovery', aqua='Mixed', orange='Understory' states. Cumulative adult *M. pyrifera* recruitment (blue) and mortality (red) and individual plant lifespans (light blue lines) are shown in panel 2. Also shown are adult *M. pyrifera* recruits (third panel), juvenile and pre-adult life stages of *M. pyrifera* (log4 counts - panel four), and stipe and adult densities of *M. pyrifera* (panel 5) at the Cardiff study site. Gold bars running through plots 2-5 indicate cohort recruitment windows, numbers indicate cohort number.

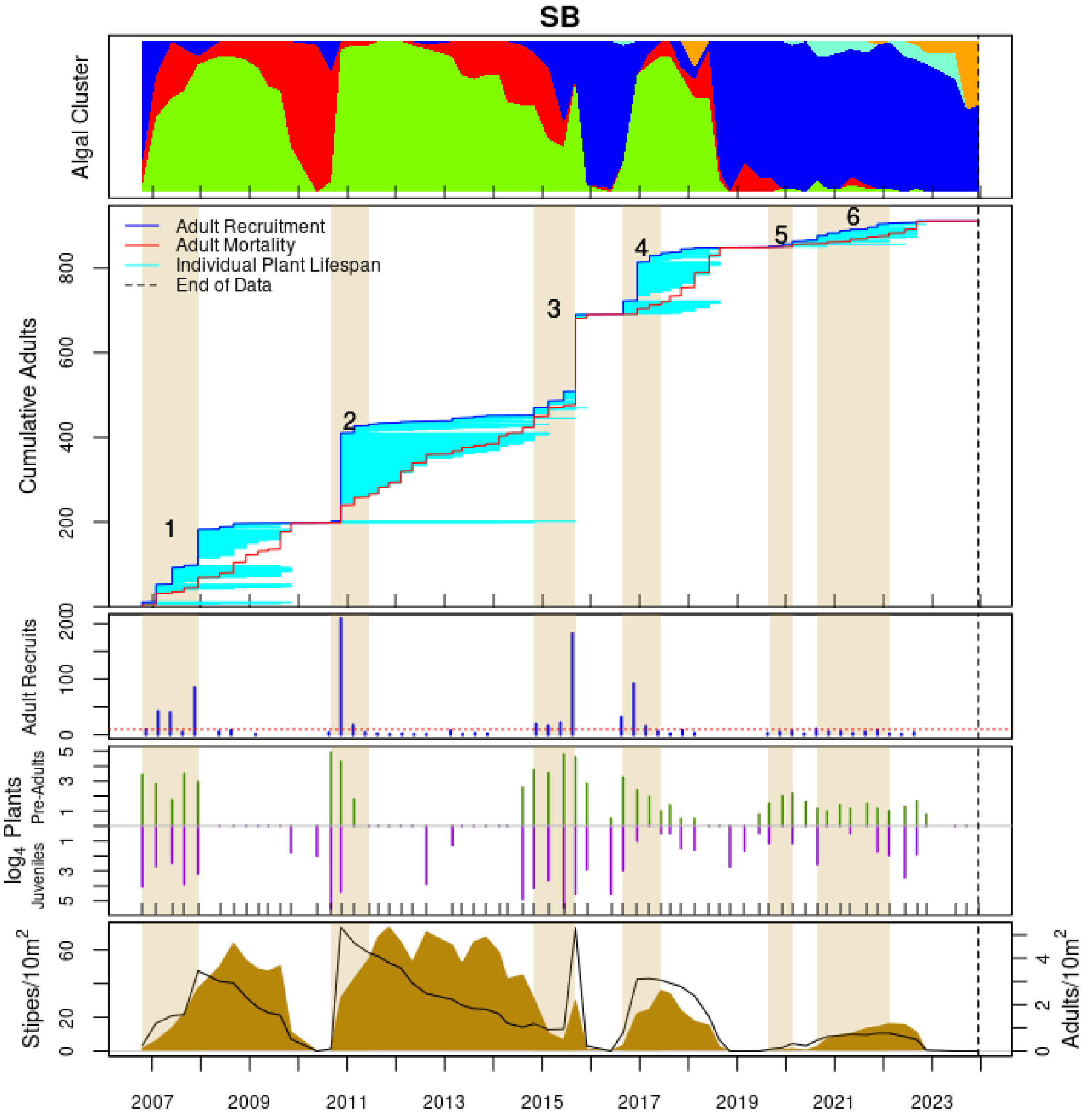

Figure S7. Same as Fig. S6 but for the Solana Beach study site.

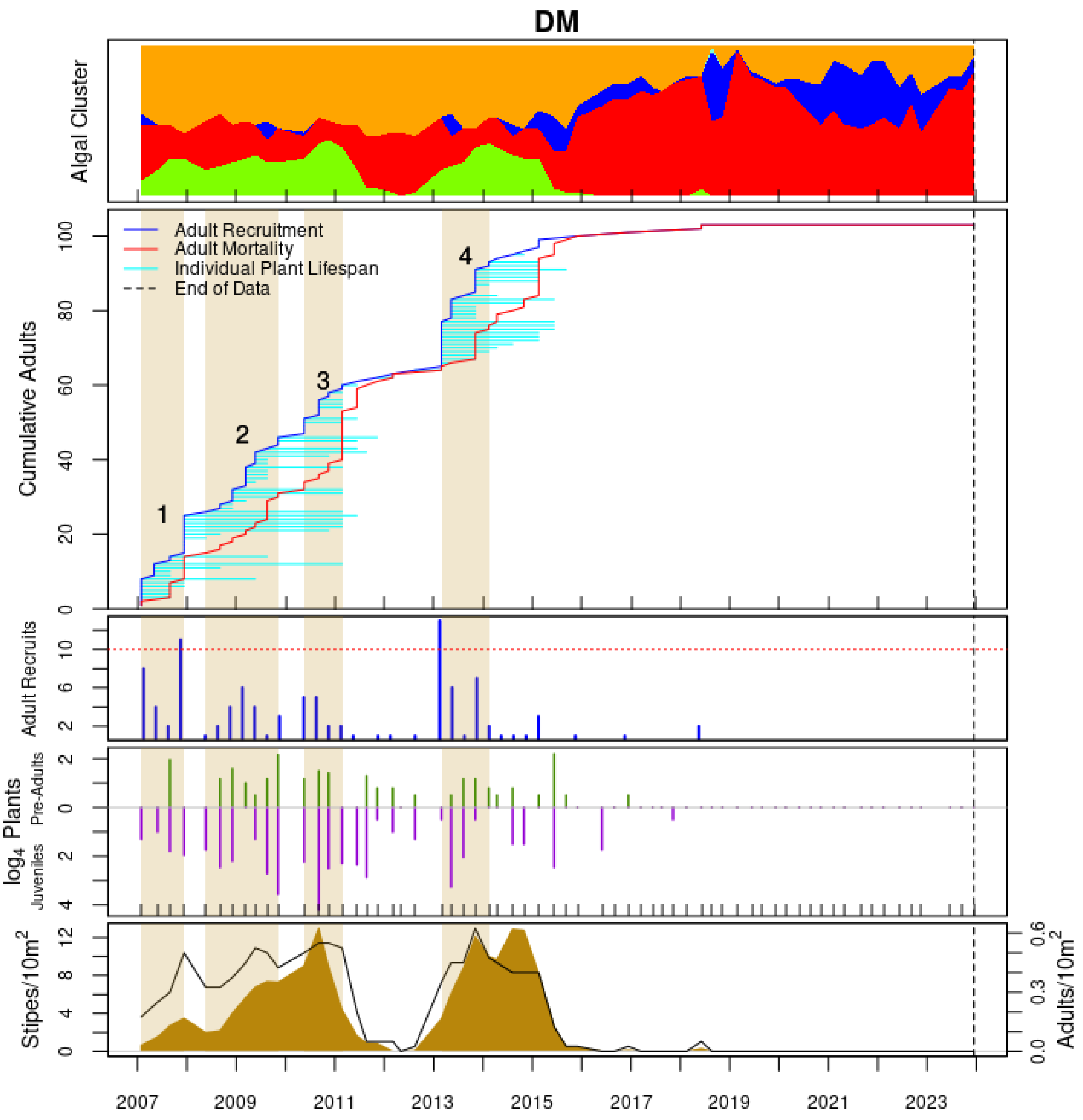

Figure S8. Same as Fig. S6 but for the Del Mar study site.

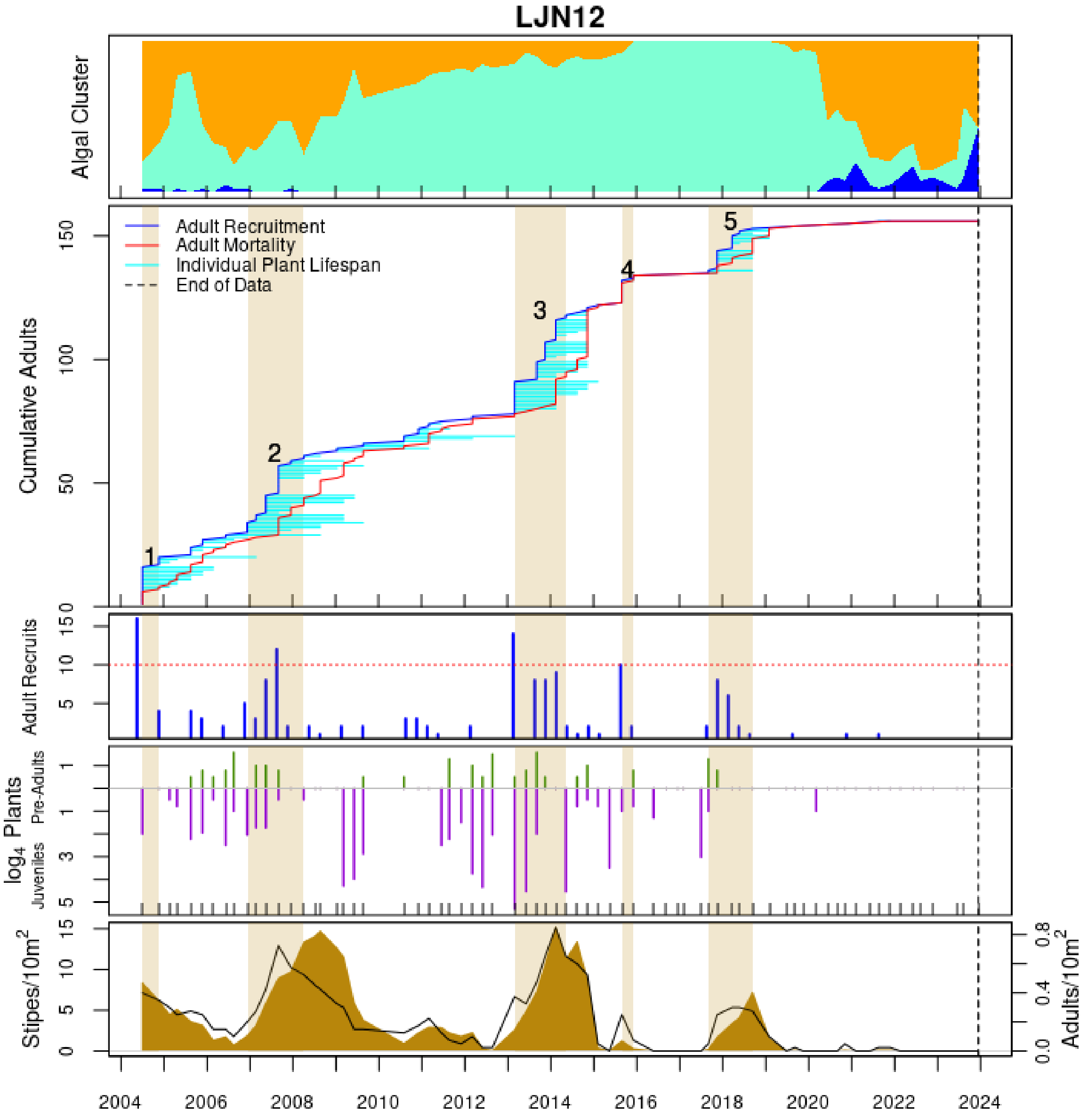

Figure S9. Same as Fig. S6 but for the LJN12 study site.

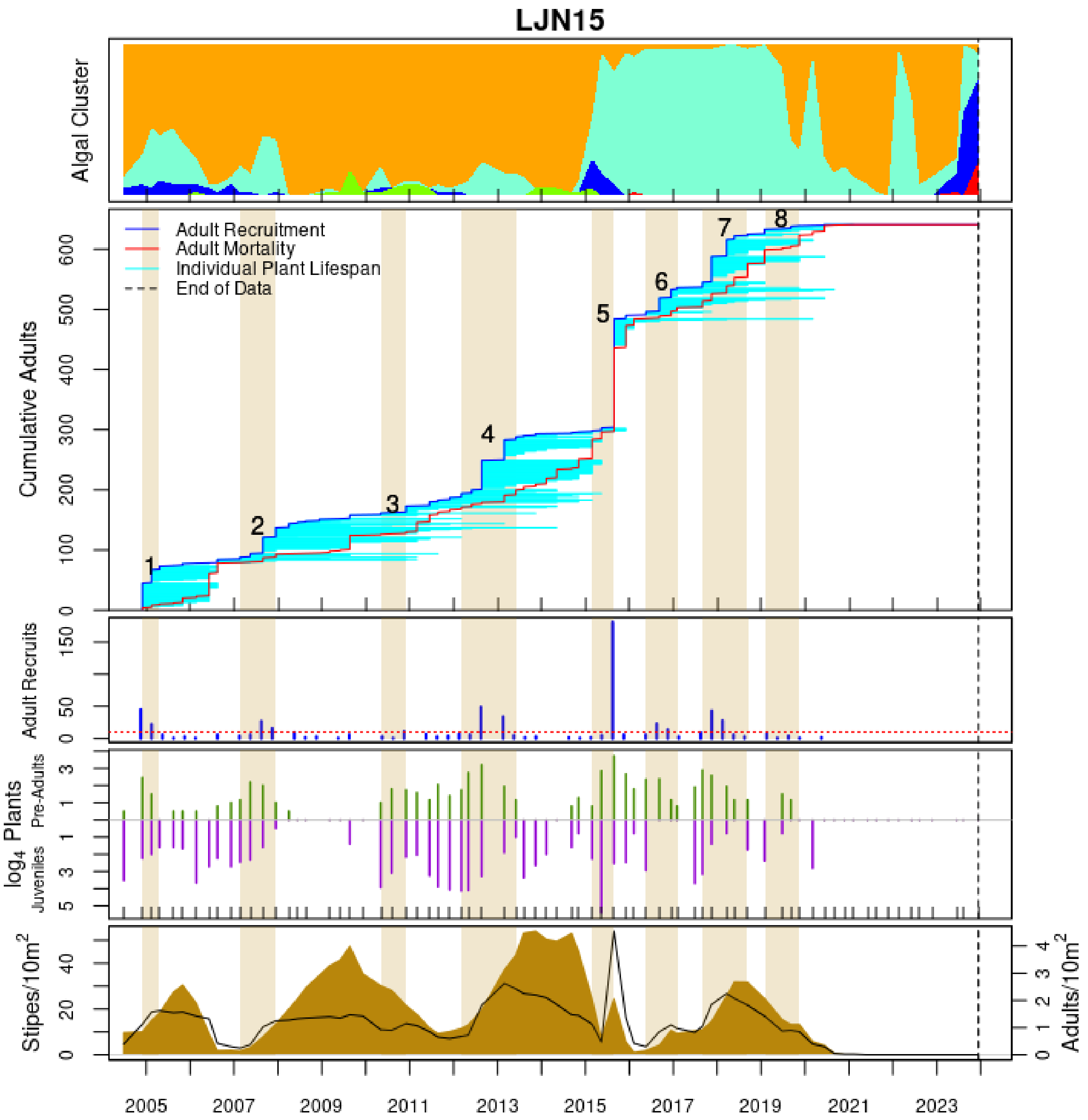

Figure S10. Same as Fig. S6 but for the LJN15 study site.

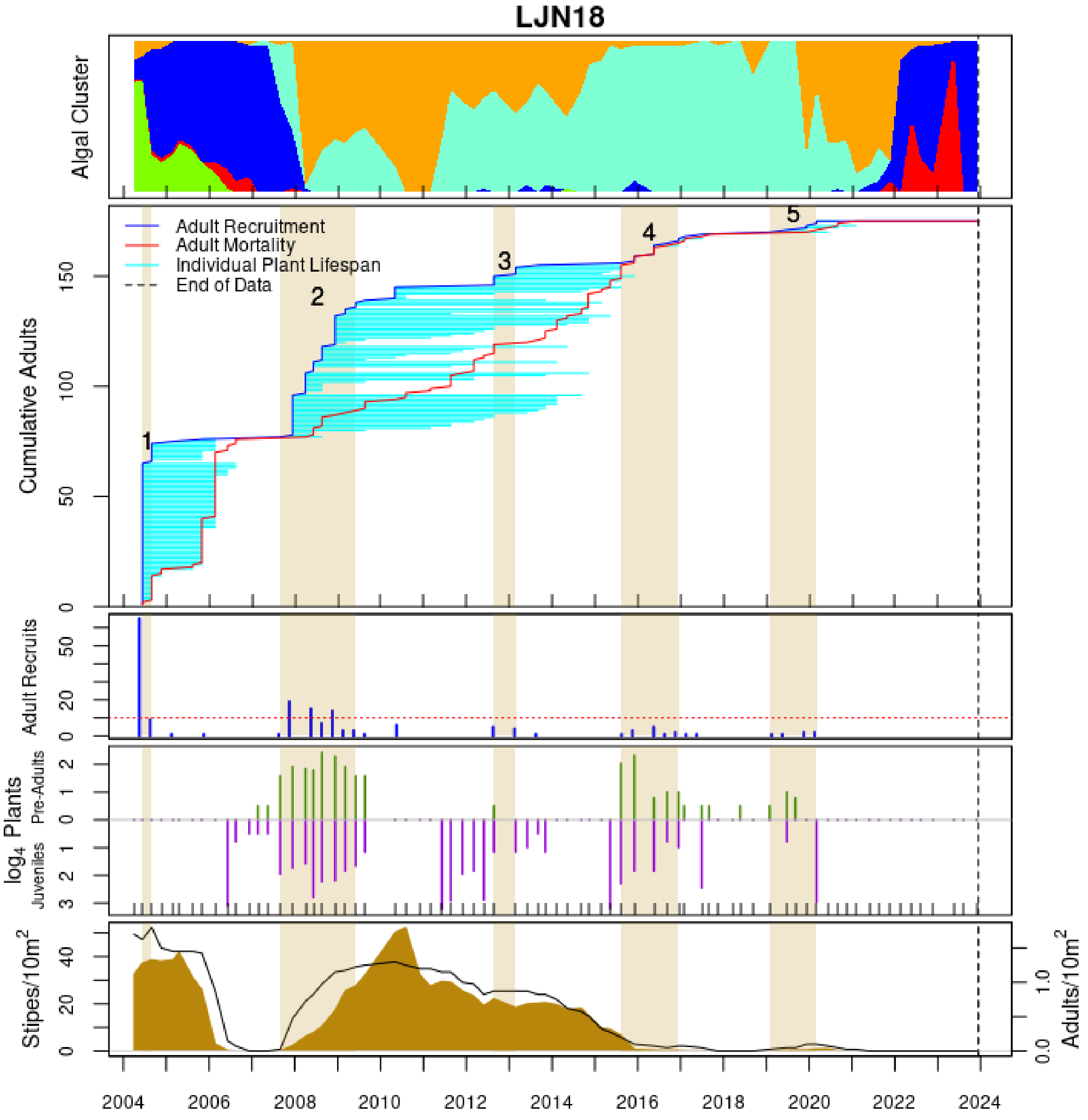

Figure S11. Same as Fig. S6 but for the LJN15 study site.

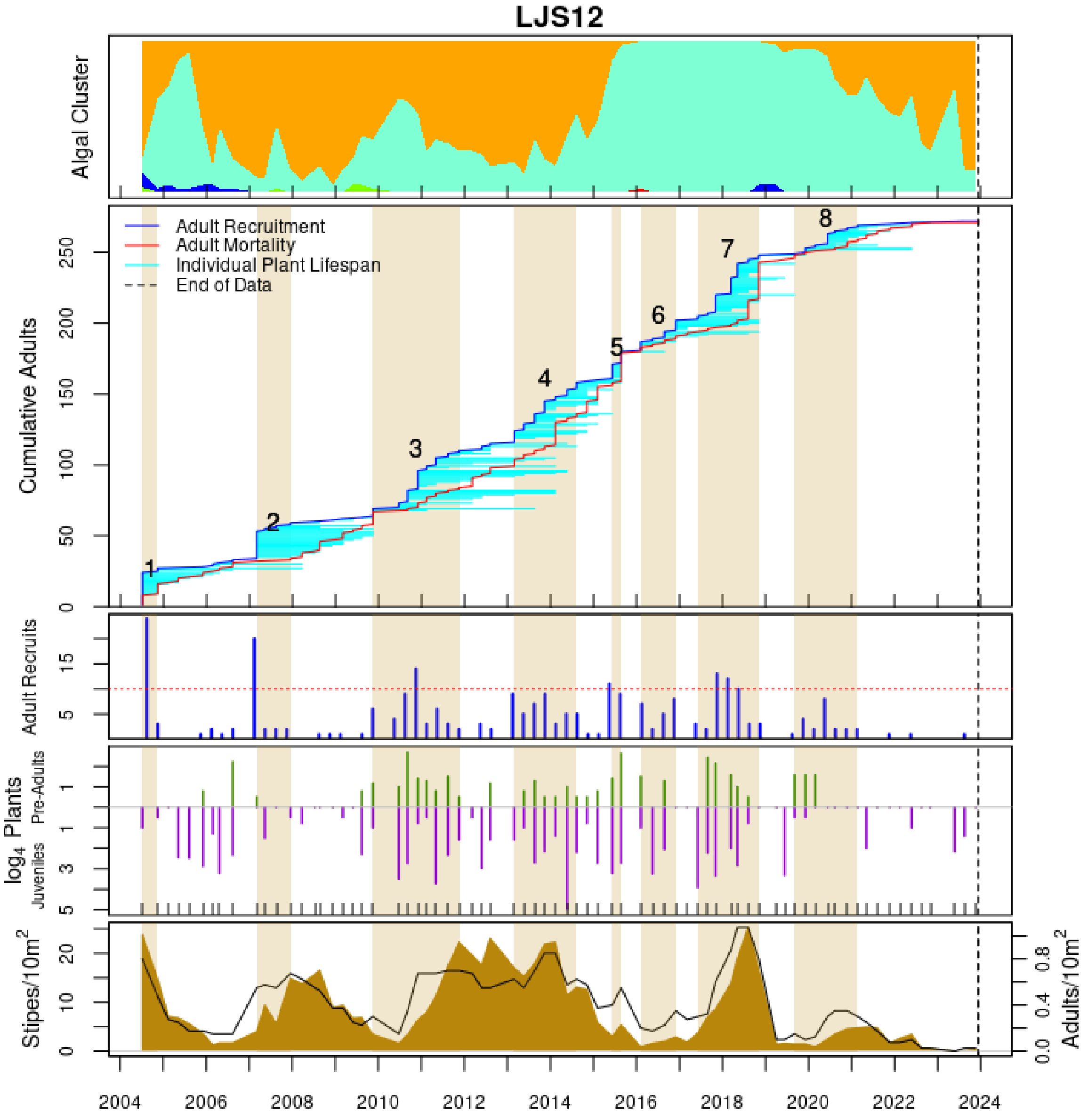

Figure S12. Same as Fig. S6 but for the LJS12 study site.

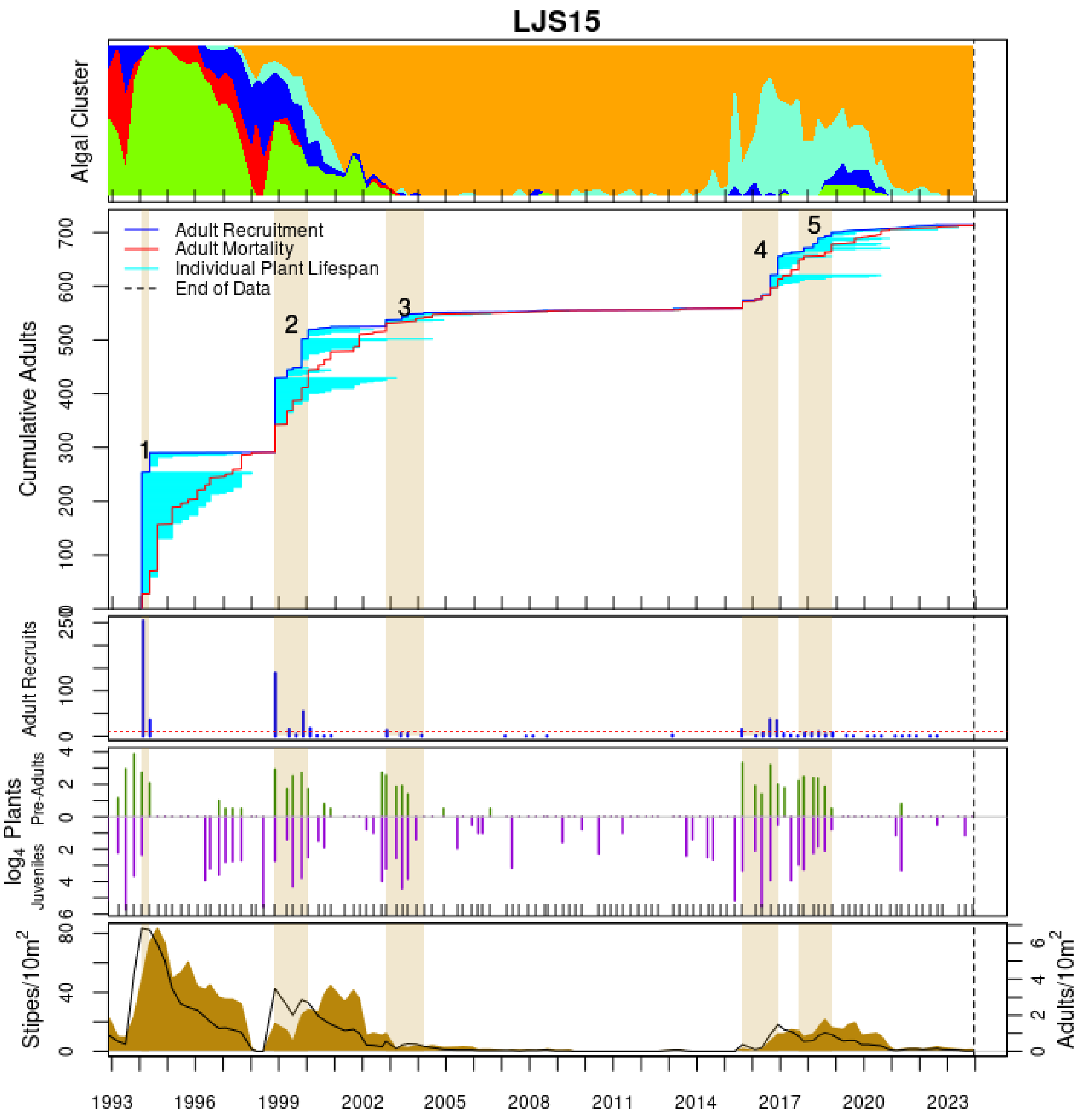

Figure S13. Same as Fig. S6 but for the LJS15 study site.

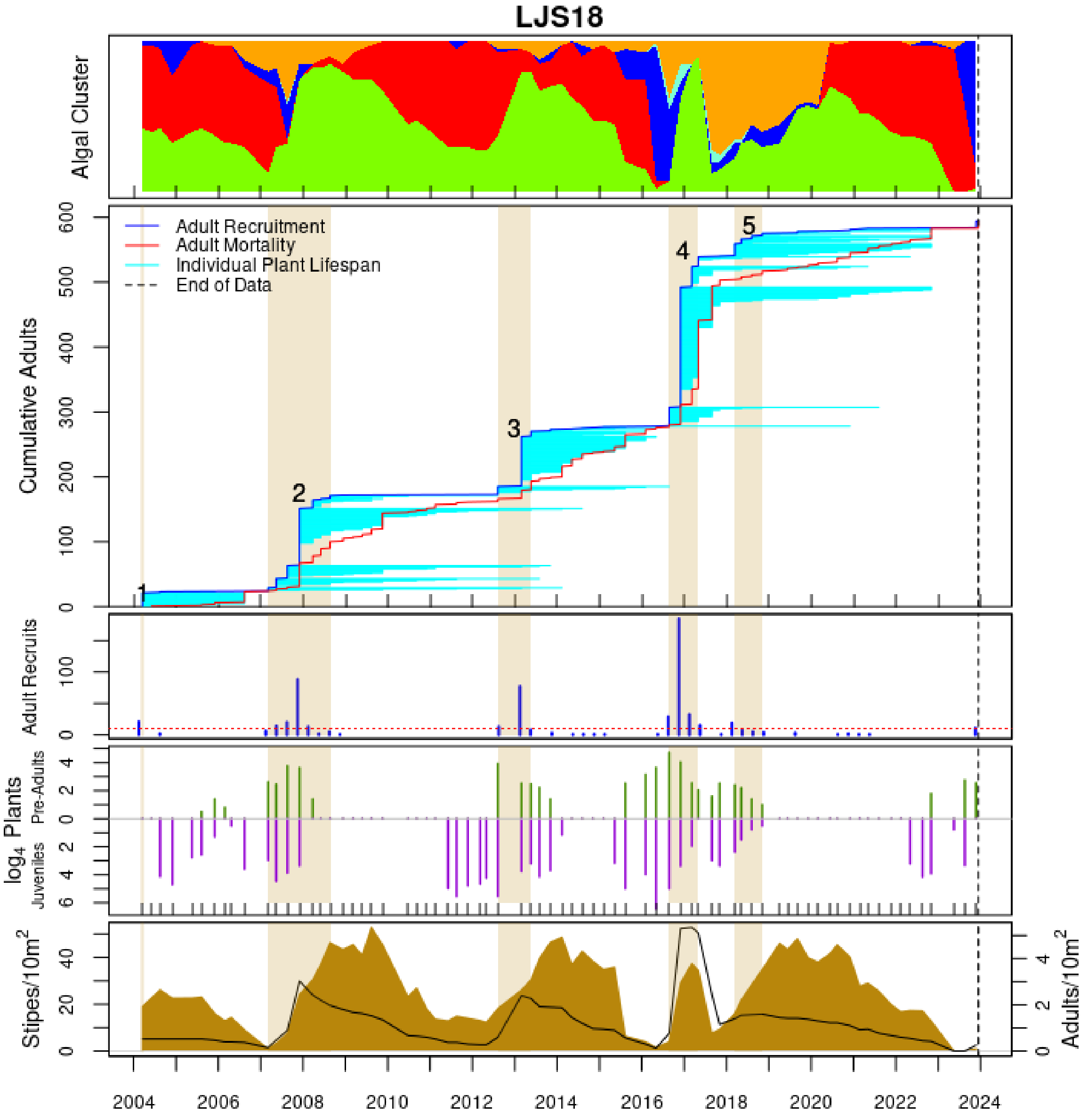

Figure S14. Same as Fig. S6 but for the LJS18 study site.

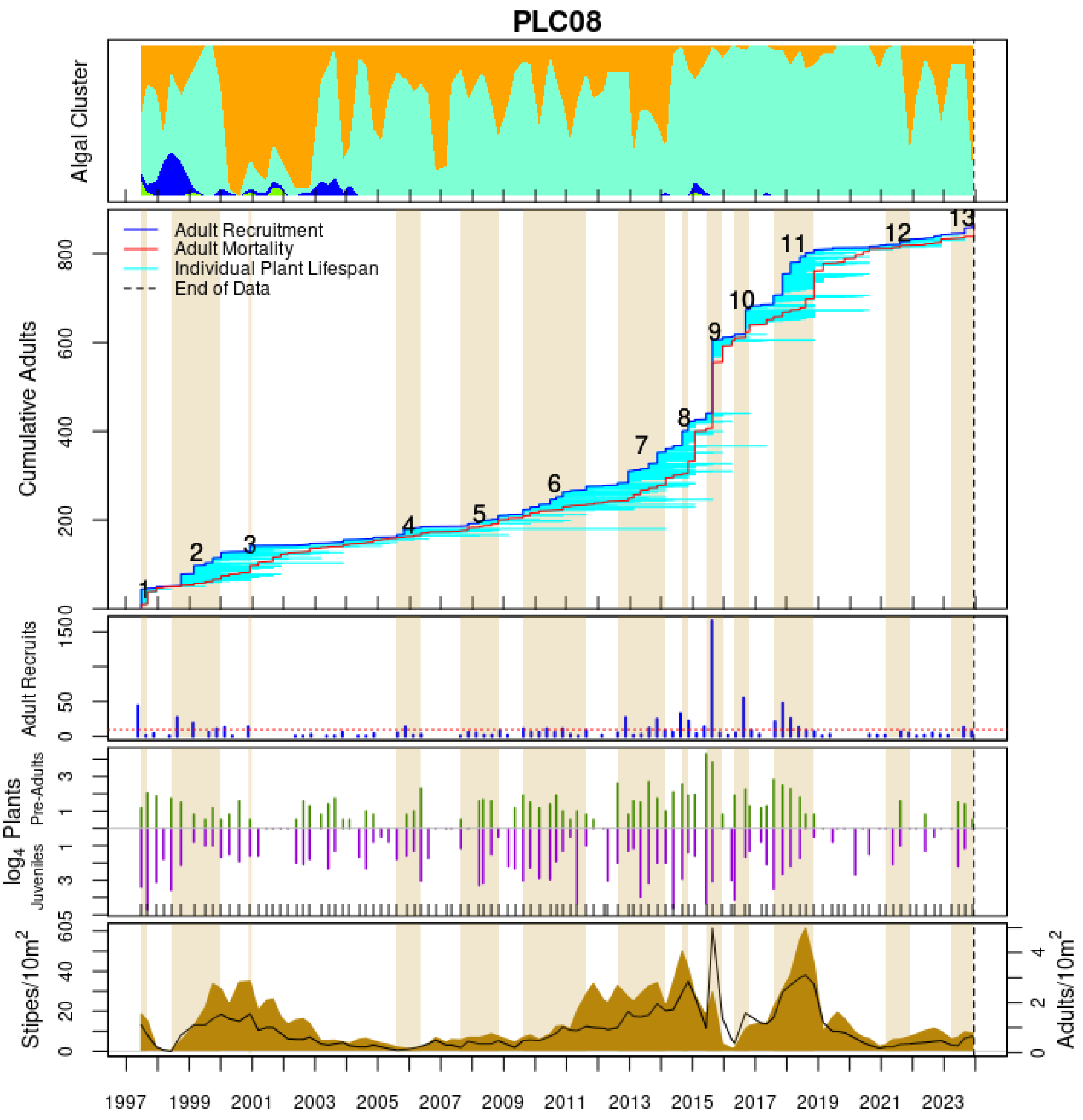

Figure S15. Same as Fig. S6 but for the PLC08 study site.

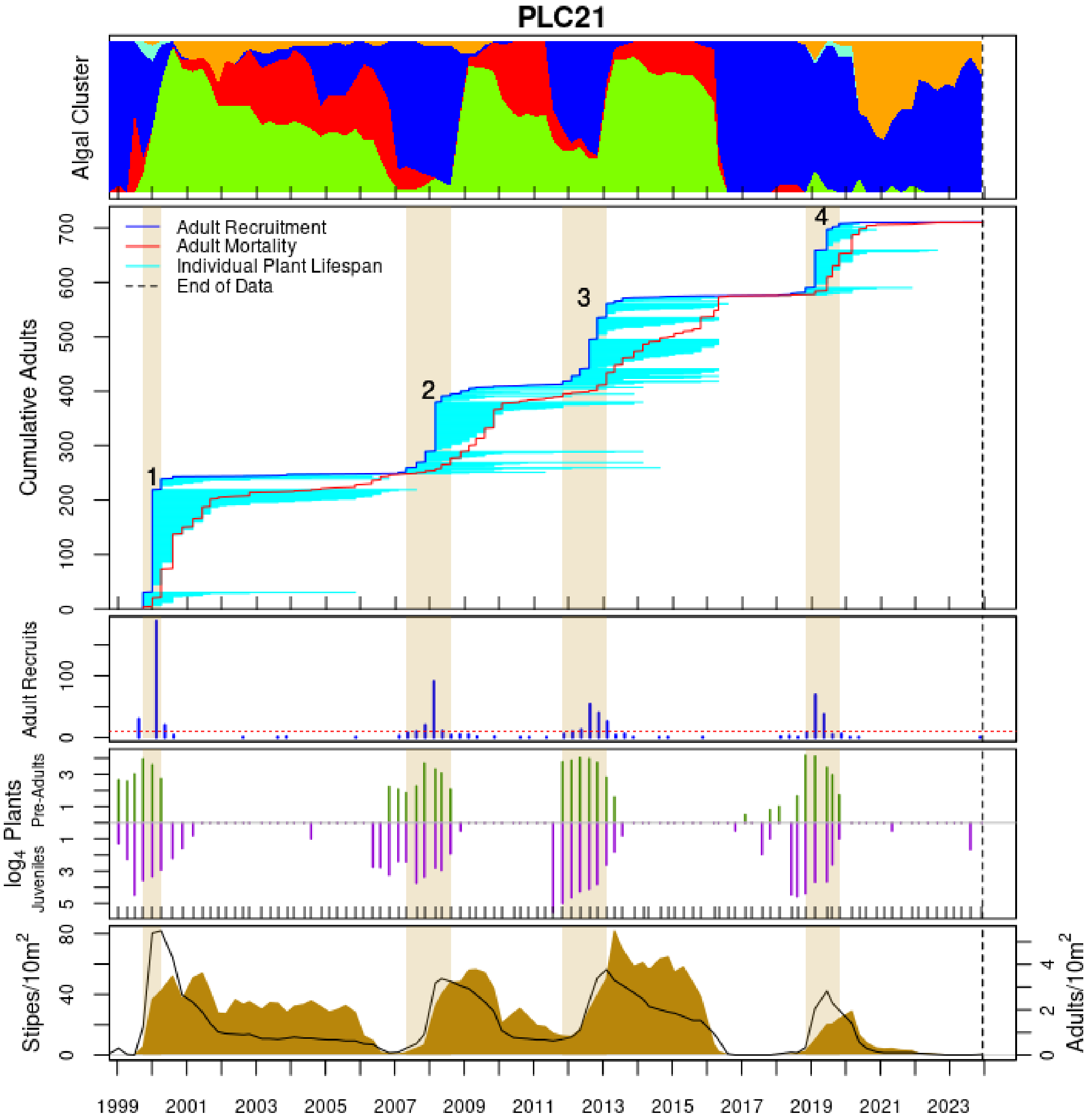

Figure S16. Same as Fig. S6 but for the PLC21 study site.

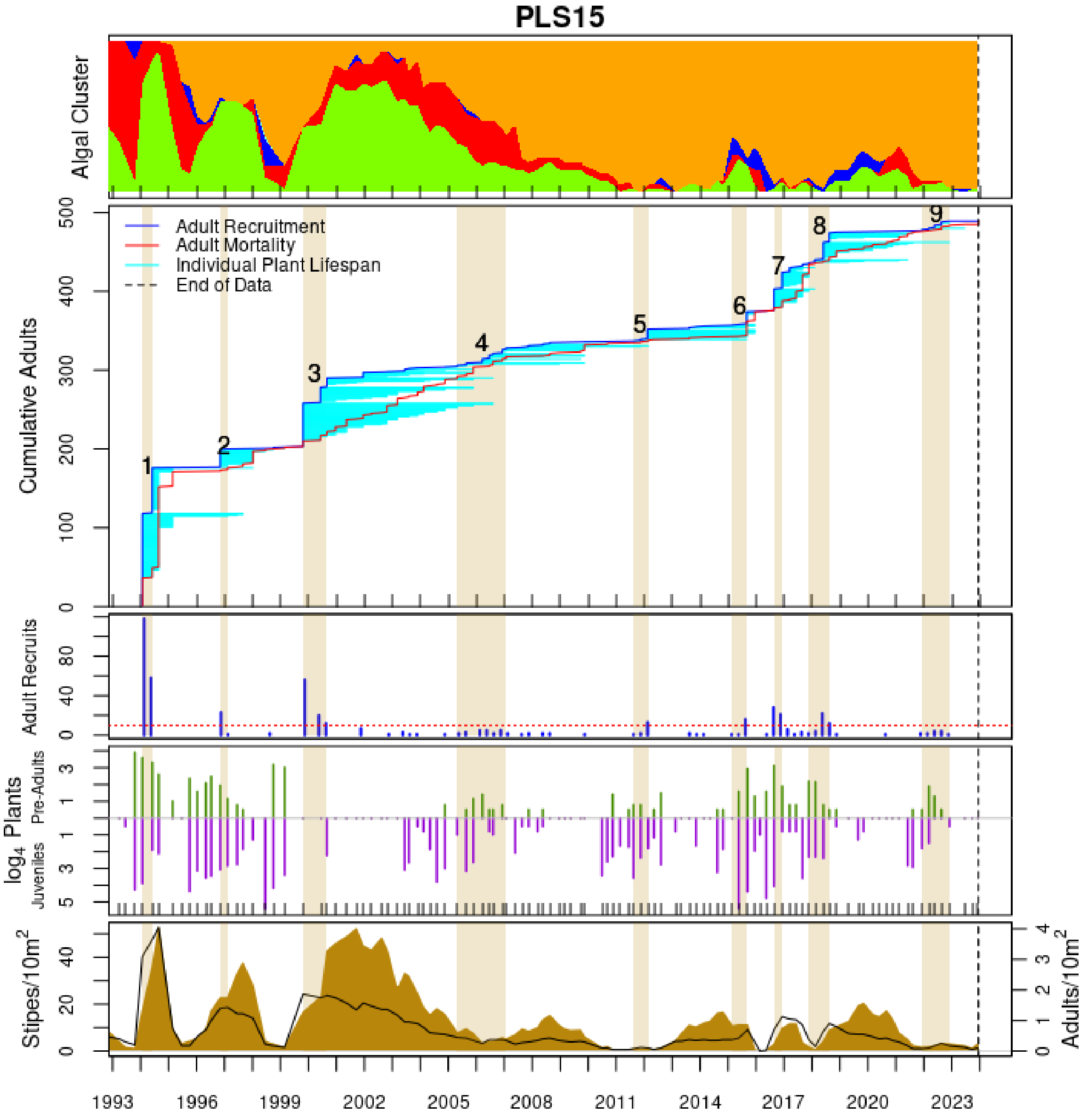

Figure S17. Same as Fig. S6 but for the PLS15 study site.

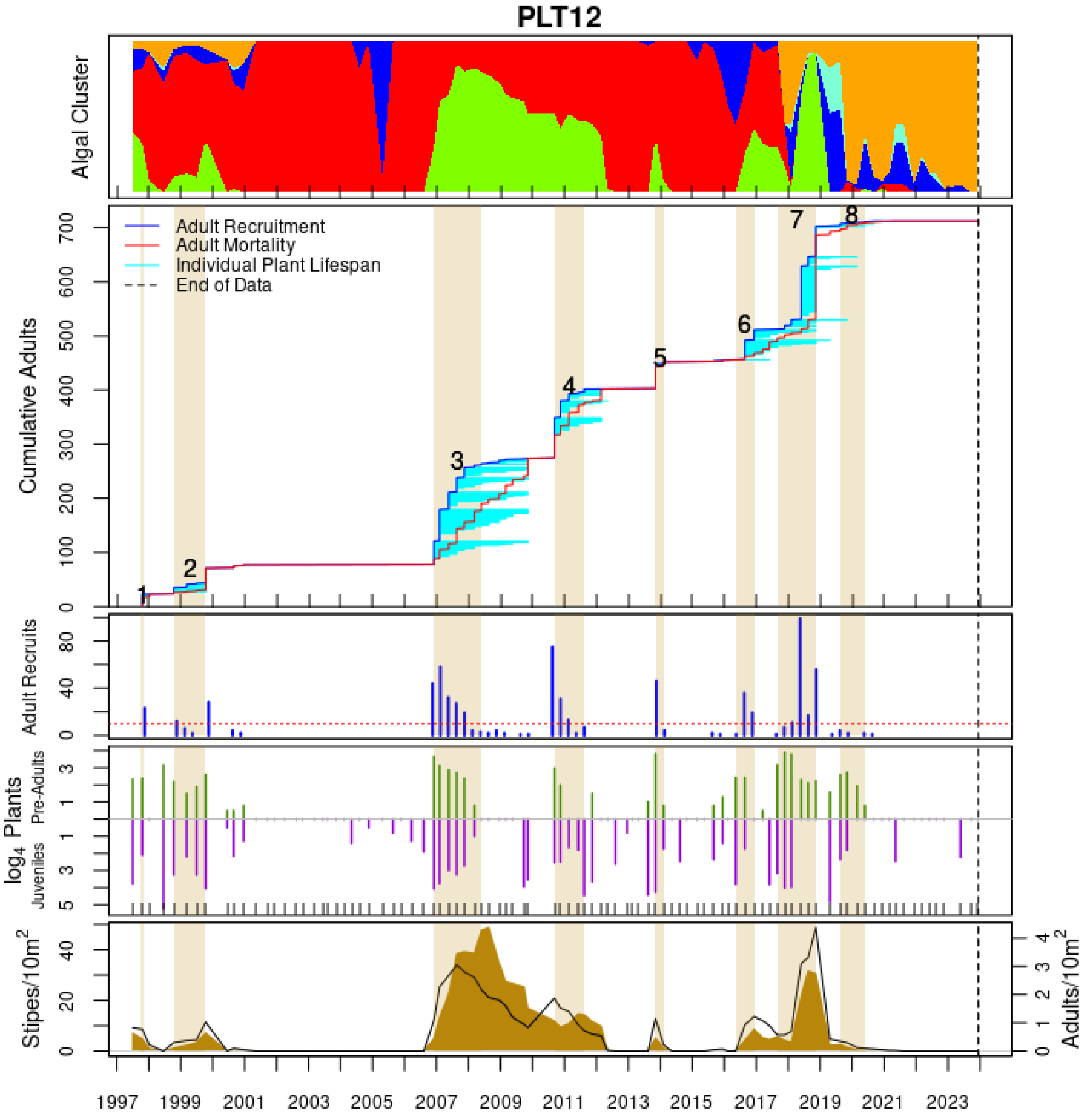

Figure S18. Same as Fig. S6 but for the PLT12 study site.

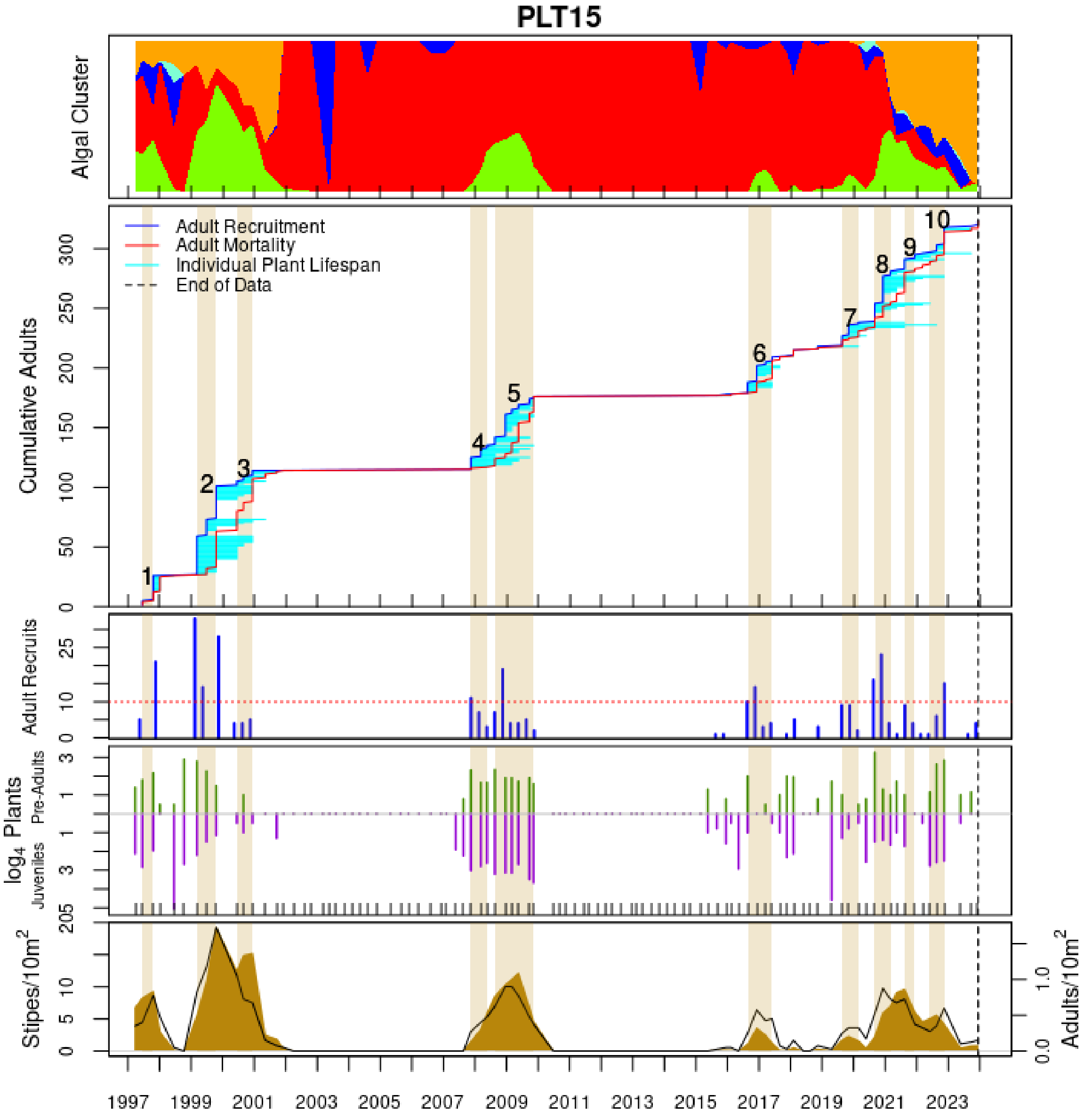

Figure S19. Same as Fig. S6 but for the PLT15 study site.

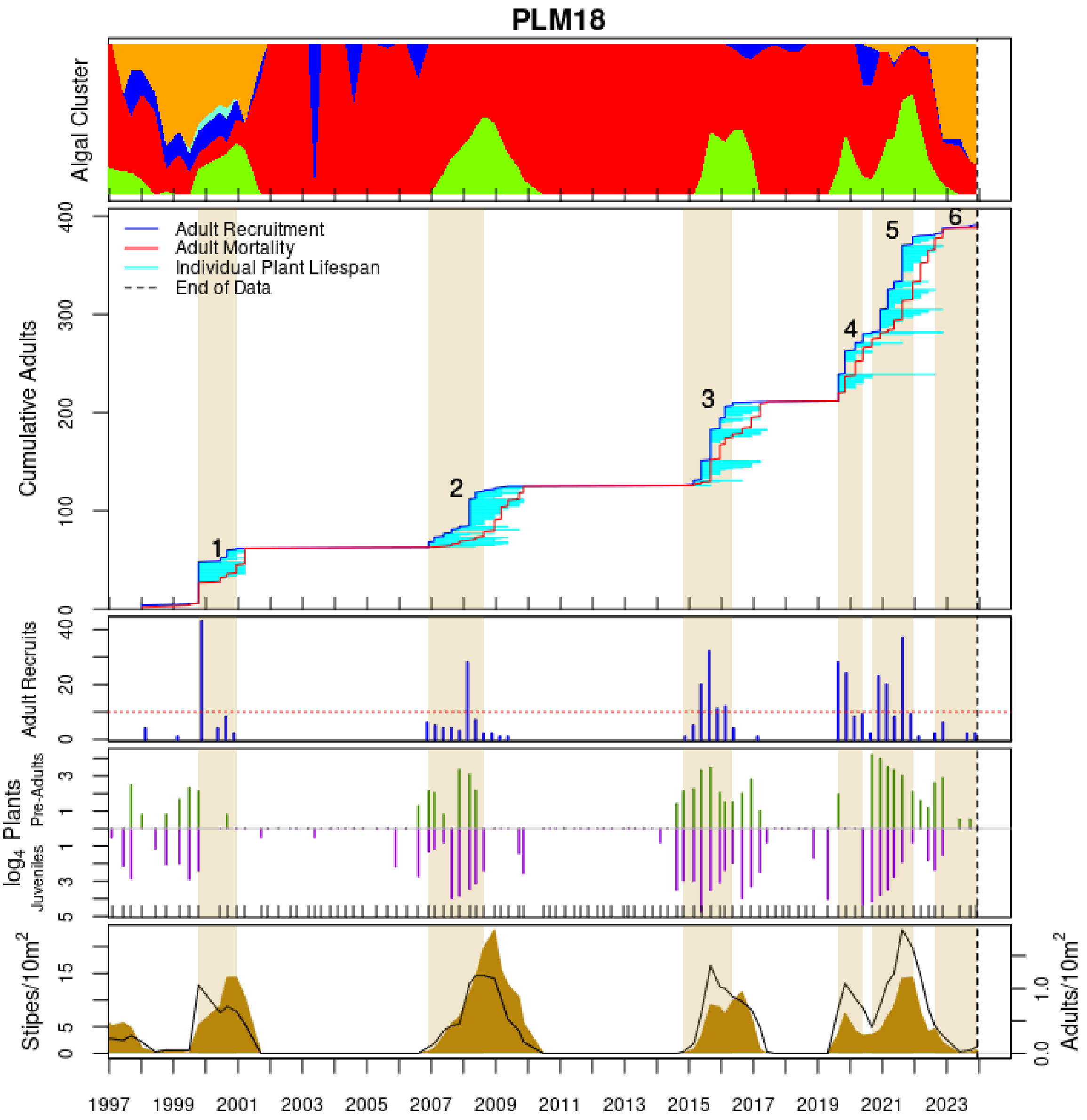

Figure S20. Same as Fig. S6 but for the PLM18 study site.
